# Effects on the Ileal Microbiota of Phosphorus and Calcium Utilization, Bird Performance, and Gender in Japanese Quail

**DOI:** 10.3390/ani10050885

**Published:** 2020-05-19

**Authors:** Daniel Borda-Molina, Christoph Roth, Angélica Hérnandez-Arriaga, Daniel Rissi, Solveig Vollmar, Markus Rodehutscord, Jörn Bennewitz, Amélia Camarinha-Silva

**Affiliations:** Institute of Animal Science, University of Hohenheim, 70599 Stuttgart, Germany; danielbm@uni-hohenheim.de (D.B.-M.); christoph.roth@uni-hohenheim.de (C.R.); angelica.hernandez@uni-hohenheim.de (A.H.-A.); daniel_rissi1@hotmail.com (D.R.); Solveig.vollmar@uni-hohenheim.de (S.V.); markus.rodehutscord@uni-hohenheim.de (M.R.); j.bennewitz@uni-hohenheim.de (J.B.)

**Keywords:** Japanese quail, ileal microbiota, phosphorus utilization, calcium utilization, gender, performance traits

## Abstract

**Simple Summary:**

The Japanese quail is an animal model for nutritional and biological studies in poultry. Diet assimilation is influenced not only by external factors, but also by the host, including its microbiota. The gut microbiota is involved in the digestion of feed constituents, facilitating the breakdown of polymers to compounds from which the animal can benefit. This study elucidates the influence of the ileal microbiota in the content of the intestine (digesta) from a large cohort of Japanese quail fed the same diet and offered identical environmental conditions. Phosphorus utilization (PU), calcium utilization, feed intake, feed conversion, and body weight gain were parameters evaluated in the birds to understand the microbial influences. A core microbial community of five bacterial species, Unc. *Lactobacillus*, Unc. Clostridaceae 1, *Clostridium* sensu stricto, *Escherichia coli*, and *Streptococcus alactolyticus*, colonized the ileum of all animals and contributed to more than 70% of the total community. Gender had a significant effect on the ileum microbial community. Even though birds were offered the same diet and housed in standardized conditions, it remains unclear if microbiota composition followed the mechanisms that caused different PU or if the change in microbiota composition and function caused the differences in PU.

**Abstract:**

In this study, we aimed to investigate the ileum digesta of a large cohort of Japanese quail fed the same diet, with similar environmental conditions. We also address how P utilization (PU), Ca utilization (CaU), and bird performance (feed intake (FI), feed conversion (FC), and body weight gain (BWG)) modify intestinal microbiota of male and female quail. Despite the great number of samples analyzed (760), a core microbiome was composed of five bacteria. The Unc. *Lactobacillus*, Unc. Clostridaceae 1, *Clostridium* sensu stricto, *Escherichia coli*, and *Streptococcus alactolyticus* were detected in all samples and contributed to more than 70% of the total community. Depending on the bird predisposition for PU, CaU, FI, BWG, and FC, those species were present in higher or lower abundances. There was a significant gender effect on the ileal microbial community. While females had higher abundances of *Lactobacillus*, males were more colonized by *Streptococcus alactolyticus*. The entire cohort was highly colonized by *Escherichia coli* (8%–15%), an enteropathogenic bacteria. It remains unclear, if microbiota composition followed the mechanisms that caused different PU, CaU, FI, FC, and BWG or if the change in microbiota composition and function caused the differences in PU, CaU, and performance traits.

## 1. Introduction

The Japanese quail (*Coturnix japonica)* is an indigenous species to Japan, China, and Korea, and it has been used as an animal model in numerous fields of poultry research in the last 60 years [1]. It was introduced as a laboratory animal in the 1960s [2] and proved to be useful in many areas of biomedical, genetics, behavior, and nutritional studies [1,3,4,5]. The short developmental period makes *C. japonica* a convenient model for biological studies. Contrarily to the broiler chicken, the quail gastrointestinal tract (GIT) has been poorly studied [6].

The microbial communities detected in the GIT of quail provide several nutritional functions to the host and play an important role in the health status of the animal [7]. Kohl et al. (2014) have described the responses of the gut microbial community to prolonged fasting in quail. Samples from colon and caeca were collected in four fasting stages (nourished, early-, mid-, and late-fasting), and the phylogenetic diversity was characterized. Fasting affected colon and cecal microbial diversity by decreasing the abundance of *Prevotella*, *Lactobacillus,* and *Faecalibacterium* [7]. Another study identified an effect of host genotype and diet on ceca microbiota [8]. Wilkinson et al. (2016) characterized the microbial community of the mouth, esophagus, crop, proventriculus, gizzard, duodenum, ileum, ceca, large intestine, and feces of eight-week-old quail (10 males and 12 females). Different microbial assemblages were observed in males and females, and ceca samples showed the highest community richness.

The dominant number of sequences found in the large intestine could not be assigned to any genera, while other detected operational taxonomic units (OTUs) belonged to the genera *Lactobacillus*, *Bacteroides*, *Ruminococcus,* and *Clostridium* [6]. In broiler chickens, gender had an influence on the microbiota composition [9].

The function of microbes in the avian gut can be distinguished into nutritional uptake, detoxification, immune-related, and the competitive exclusion of pathogens [10]. The gut microbiota is mainly involved in the digestion of feed constituents, facilitating the breakdown of polysaccharides and other molecules from which the animal can benefit. Diet composition can have a strong effect on the GIT microbiome. Variations in calcium (Ca) and phosphorus (P) supplementation altered the activity and composition of the birds’ gut microbiota [11] and performance [12].

In this study, we aimed to investigate how P utilization, Ca utilization, and bird performance (feed intake, feed conversion, and body weight gain) can modulate intestinal microbiota in male and female quail.

## 2. Materials and Methods

### 2.1. Ethical Statement

This experiment was performed in congruence with the relevant national and international laws along with the institutional guidelines. The study was approved by the animal welfare commissioner of the University of Hohenheim (approval number S371/13TE) and conducted following animal welfare regulations.

### 2.2. Sample Collection, DNA Extraction, and Illumina Library Preparation

Ileum digesta samples from 760 quail were obtained from a previous study that used an F2 design [13]. The experimental design is fully described by Beck et al. (2016). Briefly, the quails were fed with a starter diet from 1 d to 5 d (Supplementary Table S1) and then with an experimental diet (Supplementary Table S1) until the end of the experiment (15 d). Diets were designed based on the nutritional recommendations for young turkeys (Gesellschaft für Ernährungsphysiologie, 2004) [14], except for P and Ca concentration. The main feeding ingredients of the starter diet were corn, wheat, and soybean, while the experimental diet ingredients were corn, soybean, and potato protein. All information regarding phosphorus utilization (PU), calcium utilization (CaU), feed intake (FI), body weight gain (BWG), feed conversion (FC), and gender for each animal is shown in Supplementary Table S2. On day 15 of age, birds were sacrificed [15]. The ileum was longitudinally opened and digesta collected with a sterile spoon and stored in RNA later at −80 °C until further analysis. DNA was extracted using Trizol (Invitrogen, Carlsbad, CA, USA) according to the manufacturer’s instructions with a preliminary step of bead beating (30 s, 5.5 m/s) in a FastPrep instrument (MP Biomedicals, Santa Ana, CA, USA).

Library preparation was performed according to the Illumina protocol described by [16]. Briefly, primers 27F (slight modification) and 338R reported by [17,18] were used to target the V1–2 region of the 16S rRNA gene. A three-step PCR was performed using PrimeSTAR^®^ HS DNA Polymerase kit (TaKaRa, Beijing, China). The first two PCRs were prepared in a total volume of 25 µL using 1 µL of DNA template, 0.2 µM of primer, and 0.5U Taq prime start HS DNA, and the third PCR was prepared in a total volume of 50 µL. An initial denaturation at 95 °C for 3 min was followed by 10 cycles (pre and first PCR) or 20 cycles (third PCR) of denaturation at 98 °C for 10 s, annealing at 55 °C for 10 s, and an extension at 72 °C for 45 s, and then a final extension of 72 °C for 2 min. Libraries were pooled by index, standardized and purified using SequalPrep Normalization Kit (Invitrogen Inc., Carlsbad, CA, USA), and sequenced using 250 bp paired-end sequencing chemistry on an Illumina MiSeq platform.

### 2.3. Samples Grouping

The analysis of the dataset was divided into two sections, one covering the effect of PU, CaU, and animal performance on the microbial distribution (Section 1), and another on gender effects on microbiota, PU, CaU, FI, BWG, and FC (Section 2).

In the first section, three groups were created, depending on high, medium, or low predisposition for PU, CaU, FI, BWG, and FC. The high group comprised the top 50 animals, the low group contained the bottom 50 animals, and the remaining birds were grouped as medium. The groups were independently analyzed and animals may not correspond to the same birds in the different traits.

In the second section, groups were established based on the top 50 male and 50 female birds (male high and female high, respectively) and the bottom 50 male and 50 female birds (male low and female low, respectively) for PU, CaU, FI, BWG, and FC, while the remaining birds were grouped as the male or female medium. Each trait has its specific groups of males and females that may not correspond to the same birds in other traits.

### 2.4. Bioinformatics and Stratistical Analysis

Raw sequence reads obtained from Illumina MiSeq system (Illumina, Inc., San Diego, CA, USA) were analyzed using QIIME v1.9.1 pipeline (http://qiime.org/) [19], following a subsampled open-reference OTUs (operational taxonomic units) calling approach [20]. Demultiplexing and trimming of sequencing reads were done using the default parameters of the pipeline [16], with a maximum sequence length of 360 bp. The reads were merged into one fasta file and aligned using the SILVA Database (Release 132) (https://www.arb-silva.de/) [21]. Chimeras were identified and removed using usearch [22]. Reads were clustered at 97% identity into OTUs. Only OTUs present on average abundance higher than 0.0001% and with a sequence length >250 bp were considered for further analysis. The closest representative was manually identified with the seqmatch function of RDP (Ribosomal Database Project—https://rdp.cme.msu.edu/). Sequences were submitted to European Nucleotide Archive under the accession number PREJB37544.

The cut-off for bacterial taxonomy classification followed the recommendations of Yarza et al. (2014) [23]. Sample reads were standardized, and the Bray–Curtis similarity coefficient [24] was used to create a sample-similarity matrix using the (Primer 7—https://www.primer-e.com/) [25]. Permutational Multivariate Analysis of Variance (PERMANOVA) routine was used to study the significant differences and interactions between groups and PU, CaU, FI, BWG, FC, and gender (*p* < 0.05) [25].

A total of 36 birds that could not be assigned to any gender were removed from further analysis. For the visual hierarchical clustering and ordination of the community structures, a two-dimensional principal coordinate analysis (PCoA) was created, whereby the centroids representing the average plotting position of each group (high, medium, and low) of each trait PU, CaU, FI, BWG, and FC were ordinated. The differences in the microbial community structure between the different groups were identified using analysis of similarities (ANOSIM) and pair-wise comparison test [25]. Groups of samples were considered significantly different if *p*-value < 0.05. The similarity percentage analysis (SIMPER) was used to calculate the similarity between and within the groups and to identify the OTUs contributing to the observed dissimilarities [25]. The statistical differences in the abundance of specific OTUs between the groups were determined with the unpaired Welch’s *t*-test with a cut-off *p*-value < 0.05. Shannon diversity was calculated with Primer 7 software. Correlations between OTUs and traits were estimated with the Spearman coefficient using PRISM 6 (GraphPad Software, San Diego, CA, USA) and were considered significantly different if *p*-value < 0.05.

## 3. Results and Discussion

### 3.1. Effect of PU, CaU, and Animal Performance on Microbial Distribution

For the first time, ileum samples from a large cohort of Japanese quail (760 samples) were characterized regarding their microbial composition. Ileum was chosen owing to its role as the gut section of nutrient absorption and high metabolic microbial activities [6,26]. Moreover, it has been hypothesized that ileum can seed other gut sections in terms of microbial composition [6]. After removing singletons, the total number of sequences obtained from the ileum digesta of quail was 39.914.727. Sequences were clustered into 1188 OTUs and taxonomically assigned. The most abundant phylum was Firmicutes (on average (av.) 83%), followed by Proteobacteria (on av. 14%). The dominance of Firmicutes confirms previous findings from 16S rRNA gene surveys in quail ileal samples with 12 animals [6] and 160 animals [6,27]. Bacteria belonging to the Firmicutes phylum synthesize short-chain fatty acids, an energy source that is directly absorbed in the intestine [10]. Other phyla with less than 2% of relative abundance were Actinobacteria, Bacteroidetes, Epsilonproteobacteria, and Tenericutes. A total of 45 genera were detected. The six most dominant included unclassified Clostridaceae1 (on av. 29.6%), *Lactobacillus* (on av. 24%), *Escherichia-Shigella* (on av. 14%), *Clostridium* sensu stricto (on av. 14%), *Streptococcus* (on av. 8.2%), and *Enterococcus* (on av. 3.7%). These genera are known colonizers of the ileum of quail and other avian species [6,28].

The microbial community of the quail’s gastrointestinal tract has not yet been deeply analyzed, and this leads to a lack of sequencing information in the databases. As previously reported by Wilkinson et al. (2016) and other avian studies, some of the most abundant OTUs detected in the ileum could not be taxonomically classified [6,28,29]. The most abundant OTU, assigned to an unclassified Clostridiaceae1, correlated positively with PU, CaU, FI, and BWG (Supplementary Table S3). This OTU belongs to the order Clostridiales, which are known to degrade plant components, which are further fermented to short-chain fatty acids [30]. FC was negatively correlated with unclassified *Clostridium* sensu stricto (on av. 22.8%); BWG with *Streptococcus alactolyticus* (on av. 10.7%) and *Enterococcus faecium* (on av. 1.5%); PU, CaU, and FI with *Escherichia coli* (on av. 13.1%) and BWG; and FI with unclassified *Lactobacillus* (on av. 29.3%) (Supplementary Table S3). Previously positive correlations for *Lactobacillus* species with egg production and feed conversion have been reported [31]. However, in the present study, only one negative correlation was observed between a high abundant unclassified *Lactobacillus* (on av. 29.3%) and FI. The presence of *Lactobacillus* species is considered to be beneficial for the bird because they transform carbohydrates to lactic acid, inhibit pathogen adhesion to the epithelium, and decrease the pH in the ileum [12]. The pH was not measured in this study, but one hypothesis for the high abundance of *E. coli* (on av. 13%) is the increasing presence of one member of Clostridiales (unclassified Clostridiaceae1) and the non-dominance of *Lactobacillus* as indicators of a higher pH. The lower dominance of *Lactobacillus* differs from previous reports on quail [6] and broiler chicken [12]. The negative correlation between *E. faecium* and BWG contradicts the results of a previous study in broilers [32]. *E. faecium* can exert probiotic effects and enlarge the villus height in the ileum of broilers [32]. In quails, it reduced the presence of pathogens like *Salmonella* owing to the production of a bacteriocin [33].

**Figure 1:**
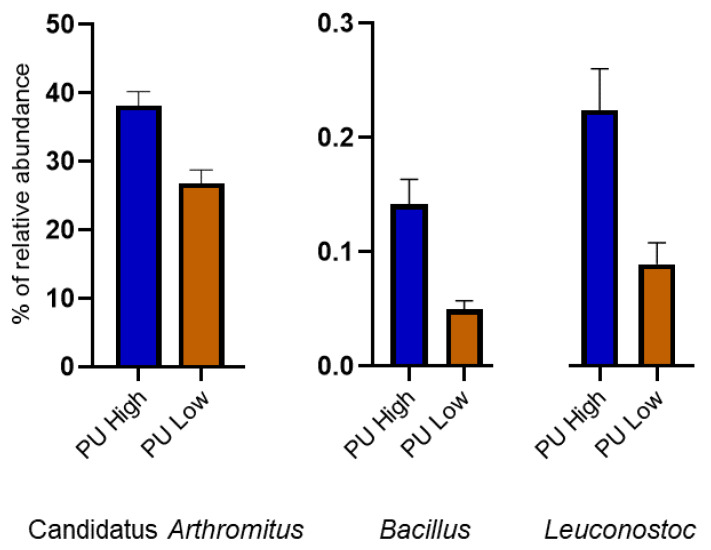
Relative abundance of the genera influenced by the P utilization (PU) in the high and low groups.

In order to better understand the effects of P and Ca utilization and other performance parameters (BWG, FI, and FC), a priori groups based on high, low, or medium bird predisposition for each trait were established. PERMANOVA test based on those a priori groups confirmed an influence of the single factors PU, CaU, and FI on the ileal microbial community (Supplementary Table S4a), while a trend was shown for the interaction BWG × FC (*p*-value < 0.10) (Supplementary Table S4b). The abundance of Candidatus *Arthromitus* was higher within birds with higher PU (Figure 1). These segmented filamentous bacteria attach to the intestine and have been previously isolated from the terminal ileum of chickens [34] and turkeys [35]. Moreover, at an early age, they have been found to positively correlate to bird performance, probably owing to its immunomodulatory capabilities [35,36]. Other genera promoted in the birds with higher PU were *Bacillus* and *Leuconostoc* (Figure 1)*. Bacillus* is considered as a probiotic in chickens; may improve bird performance [37]; exerts different enzymatic activities like amylase, xylanase, and pectinase [38]; and phosphatase activity can be expected from this genus, as previously reported in soils [39,40].

Gender had a statistically significant effect on the ileal microbial diversity of the present dataset (Supplementary Table S4c). Correspondingly, the Shannon diversity index significantly differed between males and females (Supplementary Figure S1). Previous studies demonstrated that gender differences exist in the presence of specific bacterial groups, such as *Lactobacillus* in quail [6]. In the present data set, *Lactobacillus* was more abundant in females (26% vs. 22% in males), while the abundance of *Streptococcus* tended to be the opposite (7.3% in females vs. 9.3% in males) (Supplementary Figure S2).

Considering that all birds received the same diet and were housed under the same conditions, a possible explanation for the range of performance values observed can be attributed to individual differences for diet assimilation and the presence of indigestible dietary polysaccharides [41,42]. The percentage of dissimilarity between the high, low, and medium groups for the PU, CaU, FI, BWG, and FC ranged between 52.1% and 60.9% (Supplementary Table S5). Taking into account a high individual variability not only in performance values, but also in microbial composition, it is expected that the microbial metabolic activities changed. It is possible that even bird behavior was affected as it has been demonstrated that gut microbiota affects emotional reactivity in Japanese quail [43,44].

### 3.2. Gender Effects on Microbiota, PU, CaU, FI, BWG, and FC

**Figure 2:**
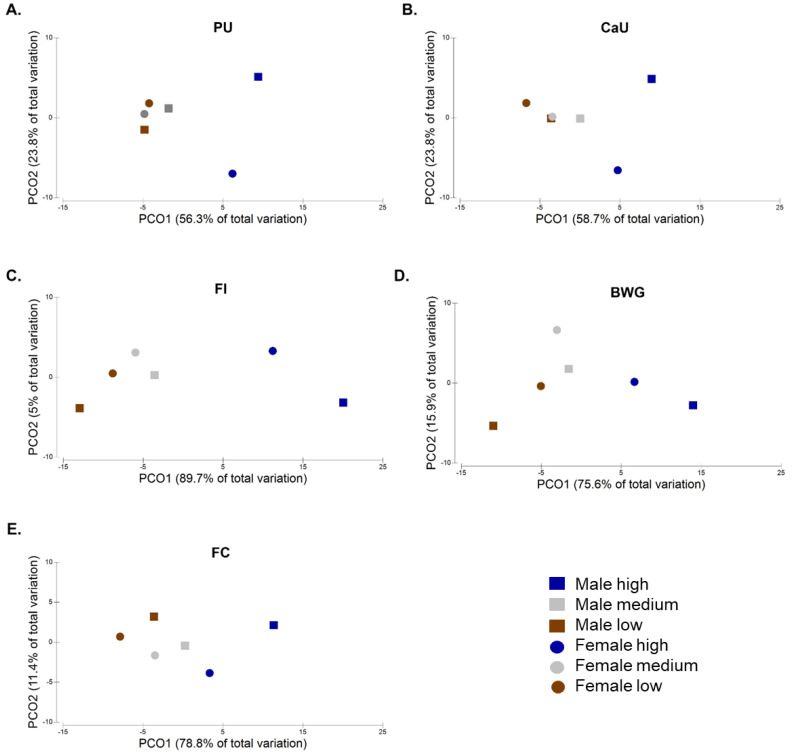
Principal coordinates analysis (PCoA) plots depicting the gender effect on (**A**) phosphorous utilization (PU), (**B**) calcium utilization (CaU), (**C**) feed intake (FI), (**D**) body weight gain (BWG), and (**E**) feed conversion (FC) in the high, medium, and low groups.

Female quail are physiologically different from males [45]; thus, it is expected to comprise different microbial resemblance. To evaluate whether gender variation exists and has an impact on PU, CaU, FI, BWG, and FC, centroids that compute the average plotting position of an a priori group of samples were calculated and ordinated using principal coordinate analysis (PCoA) (Figure 2). Gender affected the grouping of the high, medium, and low levels of PU, CaU, FI, BWG, and FC (*p*-value < 0.05). A previous study using only 200 quail observed an effect of gender on PU and CaU only as a trend [42]. It is important to highlight that, in the present study, PU ranged from 21% to 86% and CaU from 11% to 84%, a higher variation compared with that observed by Beck et al. (2014). The same study did not observe any effect of gender on FI, BWG, and FC, unlike what we observed in the present study. This discrepancy might be owing to the higher number of birds used in this study originating from an F2 design and the microbiota of the GIT being used to determine these observations.

For PU, CaU, and FI, the PCoA plots depicted three clusters comprising male/female low and medium, male high, and female high (Figure 2A–C). The two principal component axes accounted for 80% (PU), 83% (CaU), and 95% (FI) of variation among groups, thus providing a good ordination of the samples. ANOSIM pair-wise comparison tests showed a significant difference between female high versus male high, female high versus female low, and male high versus male low groups for the three traits (*p*-value < 0.05), except for the CaU between female high versus male high where a trend was observed (*p*-value = 0.06) (Supplementary Table S6). The same was not observed for female low versus male low and female medium versus male medium groups. An effect of gender in the medium group was also observed (Supplementary Table S6).

Regarding FC and BWG, the PCoA plots showed separation between low, medium, and high birds (Figure 2D,E). The two principal component axes accounted for high coverage of the total microbial variation (90% for FC and 92% for BWG). ANOSIM pairwise tests showed no statistical significance between the gender for the higher and lower group, but between high and lower groups within the same gender (*p*-value < 0.05). Regarding BWG, the female medium group was statistically different from the male medium group, while a trend was observed between the two groups for FC (*p*-value = 0.1) (Supplementary Table S6).

A group of five bacteria was responsible for the separation observed between the groups in all traits. Unclassified *Clostridiaceae1*, unclassified *Lactobacillus*, *Streptococcus alactolyticus*, unclassified *Clostridium* sensu stricto, and *Escherichia coli* contributed to more than 70% of the total community. Female and male groups were colonized by the same microorganisms, but relative abundances of microorganisms were different between genders. The average dissimilarity between the groups ranged from 51% to 62%, and the average similarity within the groups was between 37% and 50% (Supplementary Table S7).

**Figure 3:**
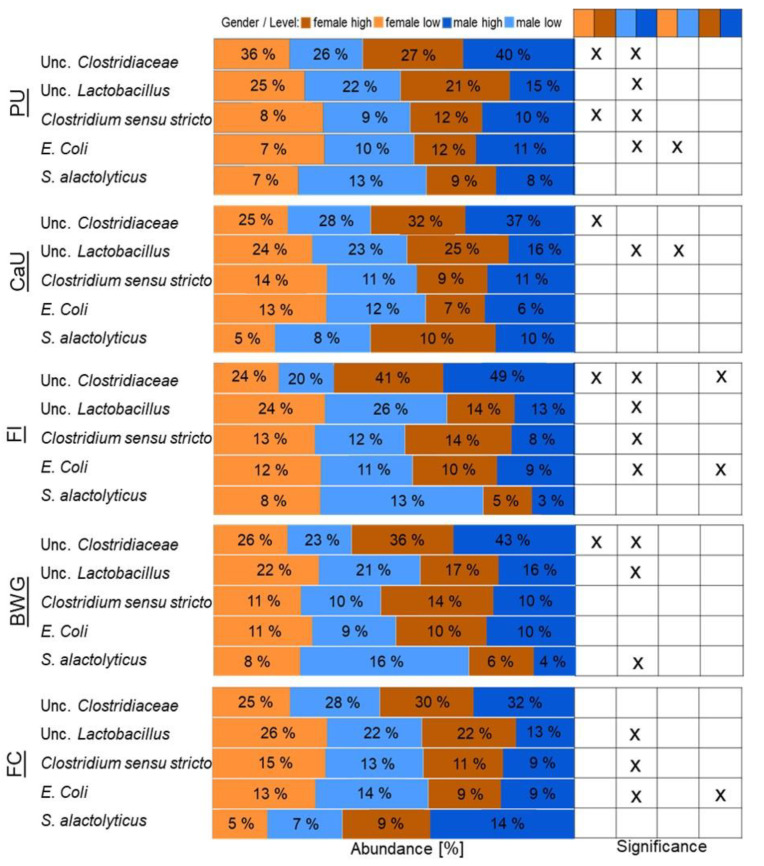
Abundance variation of the five operational taxonomic units (OTUs) that contribute to 70% of total bacterial community of females and males considering phosphorous utilization (PU), calcium utilization (CaU), feed intake (FI), body weight gain (BWG), and feed conversion (FC). Statistical significances between the groups are depicted on the graph (*p*-value < 0.05).

Pair-wise comparisons for each of the performance measurements revealed that those five bacteria abundances significantly changed based either on gender or within the gender between the high, medium, and low groups (Supplementary Table S8). Unclassified *Clostridiaceae*1 was highly abundant in the high male and female groups of all traits, with an average abundance between 32% and 49% in males and 30% and 41% in females (Figure 3 and Supplementary Table S8).

In the low female and male groups, the average abundance ranged from 20% to 28%. A significant difference in the abundance of unclassified Clostridiaceae1 was observed for PU between the groups female high versus male high (36% vs. 40%), female high versus female low (36% vs. 27%), and male high versus male low (40% vs. 26%) (*p*-value < 0.05) (Supplementary Table S8A). For the CaU, a trend was observed between the female high versus female low group (32% vs. 25%) (*p*-value < 0.06) and a statistical significance between male high and low (37% vs. 28%) (*p*-value < 0.05) (Supplementary Table S8B). In regards to feed intake, an effect was detected between female versus male high (41% vs. 49%), female high versus female low (41% vs. 24%), and male high versus male low (49% vs. 20%) (*p*-value < 0.05) (Supplementary Table S8C) and in the case of BWG between female versus male high (36% vs. 43%), female high versus female low (36% vs. 26%), and male high versus male low (43% vs. 22%) (*p*-value < 0.05) (Supplementary Table S8D). This microorganism belongs to the Clostridiales order, and it was previously detected in the gastrointestinal tract of broilers [12]. Clostridia are common colonizers of broiler and quail GIT [46] and are responsible for plant material degradation [30]. Generally, they are not the most dominant group, as observed in this study, but are detected in lower relative abundance [6,47]. Corn favored the abundance of clostridia in the avian GIT [48]. The quail of this study were fed with a corn-based diet [13], which might explain the higher abundance of the unclassified Clostridiaceae1 in the samples. Bird age has a remarkable impact on microbiota composition and diversity, gut modulation, and metabolic functions [46]. All previous studies characterizing quail GIT have worked with animals at the age of 4–8 weeks [6,47,49]. This impairs the comparison between those and the present study (two weeks old). In broiler chicken, bacterial changes during their lifespan are known to exist, with an establishment of more stable communities in older animals [46]. Regarding the quails’ GIT, there is still no knowledge of how the GIT evolves during lifespan.

*Lactobacillus* are common colonizers of the ileum of broilers and quail. They are known to improve bird health, inhibit pathogen adhesion, and maintain bacterial stability [47]. They are usually considered in the literature as beneficial; however, care should be taken because they colonize the GIT together with other species and are not independent of them. They interact either positively or negatively [12,50], and thus may have an impact on gut health. In the present study, an unclassified *Lactobacillus* was present in all traits in higher relative abundance in the low female and male groups (21%–26%) in comparison with the high groups (13%–25%) (Figure 3 and Supplementary Table S8). The female high group showed higher relative abundances (14%–25%) compared with the male group (13%–16%), while in the lower groups, the males showed higher bacterial abundance for the traits PU (22% vs. 21%) and FI (26% vs. 24%), and the females in the traits CaU (24% vs. 23%), FC (26% vs. 22%), and BWG (22% vs. 21%) (Figure 3 and Supplementary Table S8). The higher abundance of *Lactobacillus* in female birds is consistent with results by Wilkinson et al. (2016) [6], and a significant difference between gender was obtained for PU, CaU, and FCR for high and medium groups and in the medium group for FI and BWG.

*Lactobacillus* and *Streptococcus* are gram-positive lactic acid bacteria present in the GIT. Most of them are non-pathogenic and associated with host well-being. *S. alactolyticus* is a commensal bacteria that was isolated from pig intestine and chicken feces and can ferment glucose, fructose, and cellobiose [51]. *S. alactolyticus* was detected in low relative abundance in all high and low groups across all traits (3%–14% and 5%–16%, respectively). Differences between gender were detected for FC (high groups) and BWG (low groups), and within gender for PU, CaU, FI, BWG, and FC (*p*-value < 0.1). It is known that *Streptococcus* species are affected by host genotype and diet [27], but no study correlated its abundance with gender, PU, CaU, and performance traits.

Members of *Clostridium* sensu stricto are usually associated with pathogenesis and are indicators of imbalanced gut microbiota [52]. *Clostridium* sensu stricto was detected in higher abundance in the low female/male samples (9%–15%) in comparison with high female/male (8%–14%) (Figure 3 and Supplementary Table S8). An effect of gender on the abundance of *Clostridium* sensu stricto was observed for the medium groups of PU, CaU, and FC (Supplementary Table S8), where higher abundance was found in females. Despite the high abundance of this member of *Clostridium* sensu stricto, the birds of this experiment were healthy, and there was no effect on BWG, as previously suggested by (Apajalahti and Kettunen 2006).

*Escherichia coli* is an enteropathogenic bacteria that can be responsible for disease. It is a common colonizer of the avian digestive tract with no principal effect on the health status of the birds. However, it can be a potential carrier of disease to other animals and humans [53]. In this study, it was detected in a range from 10%–14% abundance in low female/male and 7%–11% in high female/male birds (Figure 3 and Supplementary Table S8). Thus, it can be hypothesized that, in comparison with chicken surveys [11,12], quail may be particularly predisposed to harbor members of the family Enterobacteriaceae, as has been reported in other studies [47]. Despite the close relative abundance between the high and low groups, statistical significance (0.05 < *p*-value < 0.1) was denoted between gender for PU (high group) and CaU (high group), with being males more colonized. Within gender, PU (female high vs. low), CaU (female high vs. low), FC (female high vs. low), and FC (male high vs. low) showed statistical significance (Supplementary Table S8).

## 4. Conclusions

Even though birds were offered the same diet and housed in similar conditions, it remains unclear if microbiota composition followed the mechanisms that caused different PU, CaU, FI, BWG, and FC, or if the change in microbiota composition and function caused the differences in PU, CaU, and performance traits. Gender affects quail gastrointestinal microbial composition and affects the distribution of specific bacterial groups. Further studies in the interplay between microbiome functionality, host physiology, gender, and genetics are necessary to uncover the real effect of minerals’ utilization and performance on microbiome distribution.

## Supplementary Materials

The following are available online at https://www.mdpi.com/2076-2615/10/5/885/s1, Figure S1: Shannon diversity index [H‘] for the overall data, based on microbial ecology resemblance for female and male Japanese quails, Figure S2: Percentage of relative abundance of the genera detected in the ileum of female and male Japanese quails, Table S1. Ingredient composition and analyzed concentrations of the diets (Adapted from Beck et al. 2014), Table S2 (excel file): Information regarding phosphorous utilization (PU), calcium utilization (CaU), feed intake (FI), body weight gain (BWG), feed conversion (FC), and gender for each animal, Table S3: Pearson correlation and its corresponding significance value of the most abundant operational taxonomic units (OTUs) against phosphorus utilization (PU), calcium utilization (CaU), feed intake (FI), body weight gain (BWG), and feed conversion (FC), Table S4: Multivariate statistical analysis for the overall data at OTU level. A. PERMANOVA analysis for P and Ca utilization. B. PERMANOVA analysis for BWG, FC, and FI. C. ANOSIM to test gender effect, Table S5: Average dissimilarity (%) between high, medium, and low groups for phosphorus utilization (PU), calcium utilization (CaU), feed intake (FI) body weight gain (BWG), and feed conversion (FC) by males and females, Table S6: ANOSIM pairwise tests by groups: phosphorus utilization (PU), calcium utilization (CaU), feed intake (FI), body weight gain (BWG), and feed conversion (FC) by males and females, Table S7 (excel file): Average similarity and dissimilarity (%) between high, medium, and low groups for phosphorus utilization (PU), calcium utilization (CaU), feed intake (FI) body weight gain (BWG), and feed conversion (FC) by males and females, Table S8: Pairwise comparison based on *t*-test for phosphorus utilization, calcium utilization, feed intake, body weight gain, and feed conversion and the most abundant OTUs (unclassified Clostridiaceae1; unclassified *Lactobacillus*; unclassified *Clostridium sensu stricto* 1; *Escherichia coli*; *Streptococcus alactolyticus*; *Enterococcus faecium*). A. Phosphorus utilization. B. Calcium utilization. C. Feed intake. D. Body weight gain. E. Feed conversion.
