# Supplementary material for: Effects on the Ileal Microbiota of Phosphorus and Calcium Utilization, Bird Performance, and Gender in Japanese Quail
Source: Animals (Basel). 2020 May 19;10(5):885. doi: 10.3390/ani10050885 (PMC7278395; doi:10.3390/ani10050885)
Supplement: Supplementary file 1 [file animals-10-00885-s001.zip › Suppl.material/suppl. material.docx]

*Supplementary Material*

P-FOWL: Effects on the ileum microbiota of phosphorus and calcium utilization, bird performance and gender in an F2 cross of Japanese quail

Daniel Borda-Molina ^1^, Christoph Roth ^1^, Angélica Hérnandez-Arriaga ^1^, Daniel Rissi ^1^, Solveig Vollmar ^1^, Markus Rodehutscord ^1^, Jörn Bennewitz ^1^ and Amélia Camarinha-Silva ^1*^

^1^ University of Hohenheim, Institute of Animal Science, 70599, Stuttgart, Germany; [amelia.silva@uni-hohenheim.de](mailto:amelia.silva@uni-hohenheim.de)

***** Correspondence: [amelia.silva@uni-hohenheim.de](mailto:amelia.silva@uni-hohenheim.de); Tel.: +49-(0)-711459-23064

**Figure S1:** Distance-based redundancy analysis (dbRDA) for A. Ca utilization (CaU), B. Feed intake (FI), C. Body weight gain (BWG), and D. Feed conversion (FC). Vectors indicate the direction of each performance trait and its relation to the groups high, edium, and low


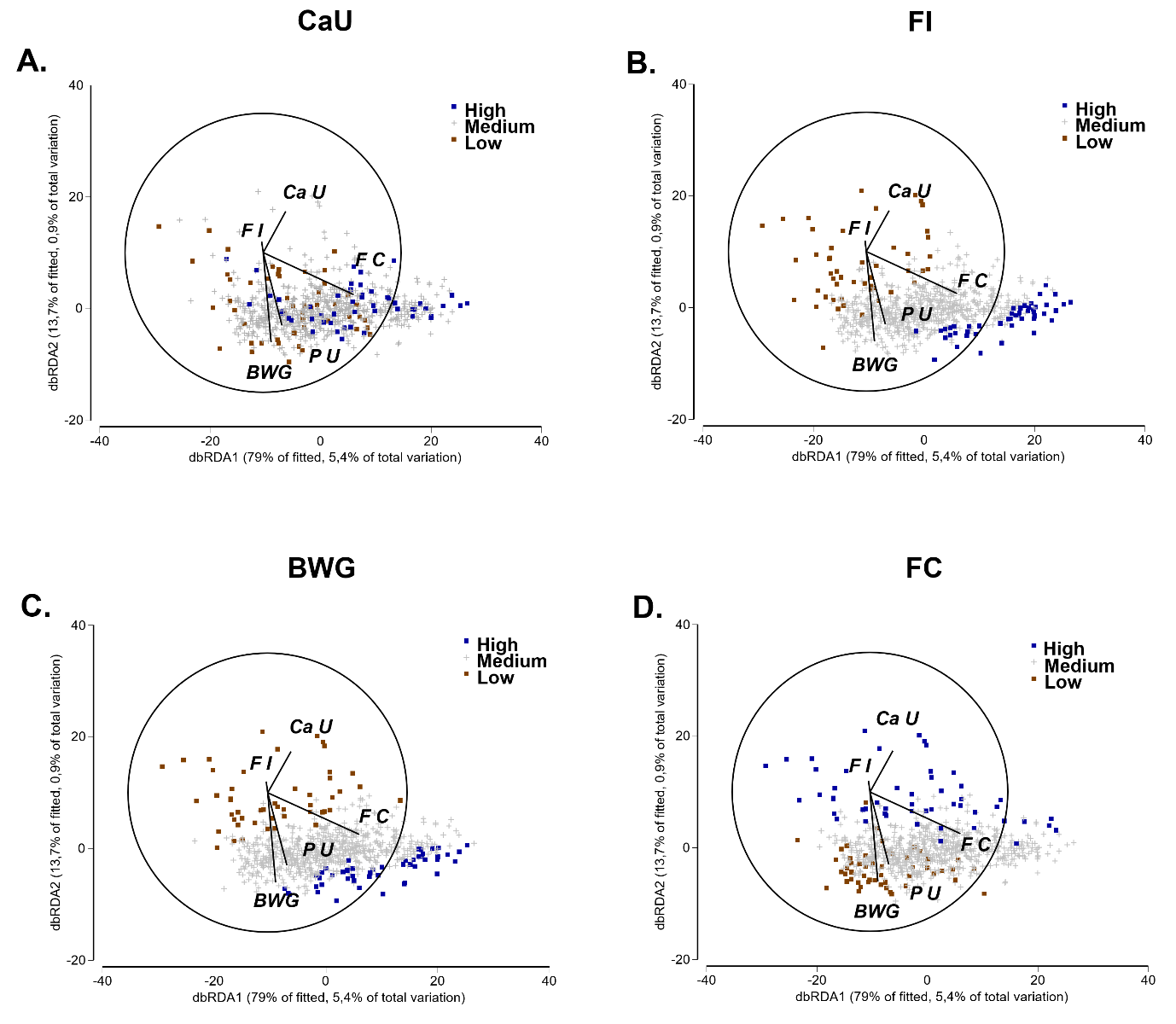


**Figure S2:** Shannon diversity index [H‘] for the overall data, based on microbial ecology resemblance for female and male Japanese quails


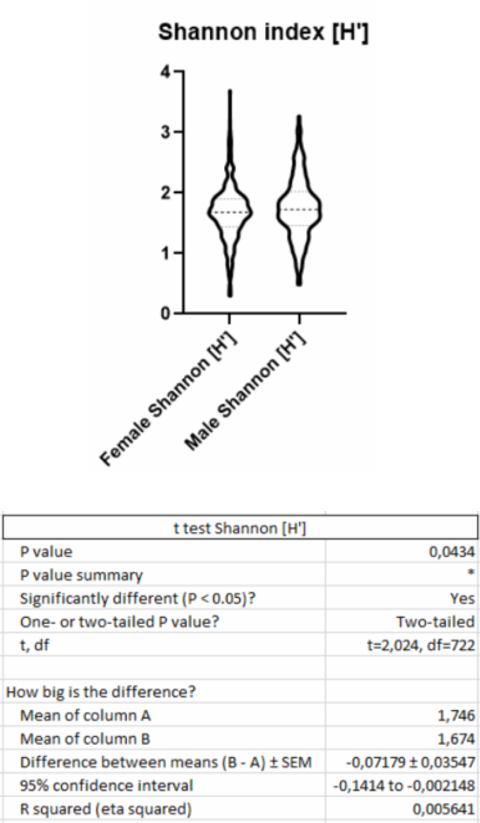


**Figure S3.** Percentage of relative abundance of the genera detected in the ileum of female and male Japanese quails


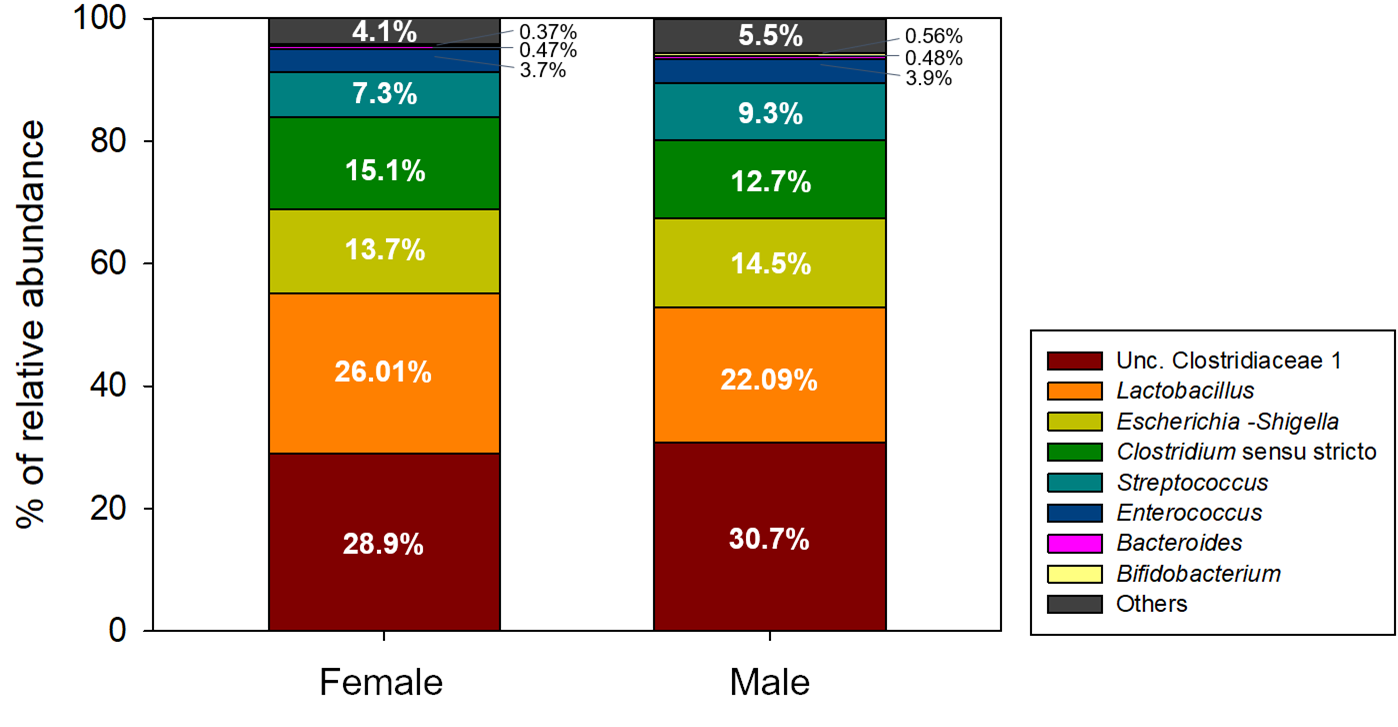


**Table S1 (excel file):** Information regarding phosphorous utilization (PU), calcium utilization (CaU), feed intake (FI), body weight gain (BWG) and feed conversion (FC), and gender for each animal.

**Table S2:** Pearson correlation and its corresponding significance value of the most abundant operational taxonomic units (OTUs) against phosphorus utilization (PU), calcium utilization (CaU), feed intake (FI), body weight gain (BWG), and Feed conversion (FC)

|  |  | PU | CaU | FI | BWG | FC |
| --- | --- | --- | --- | --- | --- | --- |
| Unclassified Clostridiaceae 1 | r | 0.176 | 0.136 | 0.341 | 0.258 | -0.037 |
|  | P-value | 1.06E-06 | 0.000 | 0.000 | 0.000 | 0.304 |
| Unclassified *Lactobacillus* | r | -0.018 | -0.047 | -0.16 | -0.07 | -0.05 |
|  | P-value | 0.610 | 0.187 | 0.000 | 0.032 | 0.139 |
| Unclassified *Clostridium* senso stricto 1 | r | -0.004 | -0.03 | -0.02 | 0.033 | -0.09 |
|  | P-value | 0.891 | 0.396 | 0.560 | 0.349 | 0.008 |
| *Escherichia coli* | r | -0.07 | -0.08 | -0.08 | -0.011 | -0.06 |
|  | P-value | 0.041 | 0.033 | 0.031 | 0.760 | 0.067 |
| *Streptococcus alactolyticus* | r | -0.06 | 0.01 | -0.06 | -0.104 | 0.086 |
|  | P-value | 0.082 | 0.666 | 0.077 | 0.004 | 0.016 |
| *Enterococcus faecium* | r | -0.03 | -0.014 | -0.06 | -0.08 | 0.05 |
|  | P-value | 0.356 | 0.684 | 0.073 | 0.016 | 0.126 |

**Table S3:** Distance-based linear model (DistLM) for defined environmental data and the microbial communities of 760 samples.

DistLM

Distance based linear models

*VARIABLES*

1 P Utilization Trial

2 Ca Utilization Trial

3 F I Trial

4 BWG Trial

5 F C Trial

Total SS(trace): 1,2848E+06

*MARGINAL TESTS*

Variable SS(trace) Pseudo-F P Prop.

P Utilization 10822 6,4385 0,0001 0,0084226

Ca Utilization 8322,9 4,9422 0,0002 0,0064778

F I 40751 24,829 0,0001 0,031717

BWG 23757 14,28 0,0001 0,01849

FC 8172,8 4,8525 0,0003 0,006361

res.df: 758

**Table S4**: Multivariate statistical analysis for the overall data at OTU level. A. PERMANOVA analysis for P and Ca utilization. B. PERMANOVA analysis for BWG, FC and FI. C. ANOSIM to test gender effect

1. PERMANOVA analysis for P and Ca utilization

Unique

Source df SS MS Pseudo-F P(perm) perms

P 4 15906 3976,4 2,3723 0,0013 9911

Ca 4 11154 2788,6 1,6637 0,0287 9903

P xCa** 4 11763 2940,7 1,7544 0,024 9915

Res 710 1,1901E+06 1676,2

Total 723 1,2364E+06

1. PERMANOVA analysis for BWG, FC and FI

Unique

Source df SS MS Pseudo-F P(perm) perms

BWG 4 7263,7 1815,9 1,1096 0,3369 9916

Feed intake 4 13694 3423,6 2,092 0,003 9912

Feed Conversion 4 6772,9 1693,2 1,0346 0,4132 9904

BWG x Feed intake 5 9381,2 1876,2 1,1465 0,2746 9893

BWG x Feed Conversion 7 15596 2228 1,3614 0,0755 9881

Feed intake x Feed Conversion 8 13081 1635,2 0,99918 0,4699 9883

BWG x Feed int. x Feed Conv. 0 0 No test

Residuals 690 1,1292E+06 1636,5

Total 723 1,2364E+06

1. ANOSIM to test gender effect

Analysis of Similarities

One-Way - A

*Tests for differences between unordered Gender groups*

*Global Test*

Sample statistic (R): 0,005

Significance level of sample statistic: 1,3%

Number of permutations: 9999 (Random sample from a large number)

Number of permuted statistics greater than or equal to R: 131

**Table S5:** Average dissimilarity (%) between high, medium and low groups for phosphorus utilization (PU), calcium utilization (CaU), feed intake (FI) body weight gain (BWG) and feed conversion (FC) by males and females

|  | High vs. Medium | Low vs. Medium | High vs. Low |
| --- | --- | --- | --- |
| PU | 54.6 | 57.9 | 58.6 |
| CaU | 55.2 | 54.6 | 54.6 |
| FI | 56.4 | 57.3 | 60.9 |
| BWG | 55.4 | 58.9 | 60.4 |
| FC | 60.3 | 52.1 | 58.4 |

**Table S6:** ANOSIM pairwise tests by groups: phosphorus utilization (PU), calcium utilization (CaU), feed intake (FI), body weight gain (BWG), and feed conversion (FC)) by males and females

| Anosim |  | male high - male low | male high - female high | male medium - female medium | male low - female low | female high - female low |
| --- | --- | --- | --- | --- | --- | --- |
| PU | R-statistic | 0.048 | 0.032 | 0.006 | -0.009 | 0.03 |
|  | p-value | 0.005 | 0.024 | 0.026 | 0.8 | 0.023 |
| CaU | R-statistic | 0.038 | 0.024 | 0.005 | 0.002 | 0.03 |
|  | p-value | 0.01 | 0.06 | 0.028 | 0.342 | 0.027 |
| FI | R-statistic | 0.255 | 0.028 | 0.007 | -0.009 | 0.092 |
|  | p-value | 0.0001 | 0.035 | 0.012 | 0.762 | 0.0001 |
| BWG | R-statistic | 0.133 | 0.011 | 0.007 | 0 | 0.029 |
|  | p-value | 0.0001 | 0.156 | 0.018 | 0.43 | 0.021 |
| FC | R-statistic | 0.06 | 0.004 | 0.003 | -0.002 | 0.027 |
|  | p-value | 0.002 | 0.305 | 0.1 | 0.497 | 0.027 |

**Table S7 (excel file)**: Average- similarity and dissimilarity (%) between high, medium and low groups for phosphorus utilization (PU), calcium utilization (CaU), feed intake (FI) body weight gain (BWG) and feed conversion (FC) by males and females

**Table S8**: Pairwise comparison based on t-test for phosphorus utilization, calcium utilization, feed intake, body weight gain, and feed conversion and the most abundant OTUs (Unclassified Clostridiaceae1; Unclassified *Lactobacillus*; Unclassified *Clostridium sensu stricto* 1; *Escherichia coli*; *Streptococcus alactolyticus*; *Enterococcus faecium*). A. Phosphorus utilization. B. Calcium utilization. C. Feed intake. D. Body weight gain. E. Feed conversion.

1. **Phosphorus utilization**

**Unclassified Clostridiaceae1**

| **PU Gender** | **Estimate** | **Std Error** | **DF** | **Lower 95%** | **Upper 95%** | **Arithmetic Mean Estimate** | **N** |
| --- | --- | --- | --- | --- | --- | --- | --- |
| PU_female_high | 35.894688 | 2.5983536 | 753 | 30.793809 | 40.995566 | 35.894688 | 50 |
| PU_female_low | 27.124174 | 2.5983536 | 753 | 22.023296 | 32.225053 | 27.124174 | 50 |
| PU_female_medium | 27.587116 | 1.1372685 | 753 | 25.354522 | 29.819710 | 27.587116 | 261 |
| PU_male_high | 40.086446 | 2.5983536 | 753 | 34.985568 | 45.187325 | 40.086446 | 50 |
| PU_male_low | 26.092141 | 2.5983536 | 753 | 20.991263 | 31.193019 | 26.092141 | 50 |
| PU_male_medium | 29.512019 | 1.1329360 | 753 | 27.287931 | 31.736108 | 29.512019 | 263 |

| **PU Gender** | **-PU Gender** | **Difference** | **Std Error** | **t Ratio** | **Prob>\|t\|** | **Lower 95%** | **Upper 95%** |
| --- | --- | --- | --- | --- | --- | --- | --- |
| PU_female_high | PU_female_low | 8.7705 | 3.674627 | 2.39 | 0.0172* | 1.5568 | 15.9842 |
| PU_female_high | PU_female_medium | 8.3076 | 2.836339 | 2.93 | 0.0035* | 2.7395 | 13.8756 |
| PU_female_high | PU_male_high | -4.1918 | 3.674627 | -1.14 | 0.2543 | -11.4055 | 3.0220 |
| PU_female_high | PU_male_low | 9.8025 | 3.674627 | 2.67 | 0.0078* | 2.5888 | 17.0163 |
| PU_female_high | PU_male_medium | 6.3827 | 2.834605 | 2.25 | 0.0246* | 0.8180 | 11.9473 |
| PU_female_low | PU_female_medium | -0.4629 | 2.836339 | -0.16 | 0.8704 | -6.0310 | 5.1051 |
| PU_female_low | PU_male_high | -12.9623 | 3.674627 | -3.53 | 0.0004* | -20.1760 | -5.7485 |
| PU_female_low | PU_male_low | 1.0320 | 3.674627 | 0.28 | 0.7789 | -6.1817 | 8.2458 |
| PU_female_low | PU_male_medium | -2.3878 | 2.834605 | -0.84 | 0.3998 | -7.9525 | 3.1768 |
| PU_female_medium | PU_male_high | -12.4993 | 2.836339 | -4.41 | <.0001* | -18.0674 | -6.9313 |
| PU_female_medium | PU_male_low | 1.4950 | 2.836339 | 0.53 | 0.5983 | -4.0731 | 7.0630 |
| PU_female_medium | PU_male_medium | -1.9249 | 1.605280 | -1.20 | 0.2309 | -5.0763 | 1.2265 |
| PU_male_high | PU_male_low | 13.9943 | 3.674627 | 3.81 | 0.0002* | 6.7806 | 21.2080 |
| PU_male_high | PU_male_medium | 10.5744 | 2.834605 | 3.73 | 0.0002* | 5.0098 | 16.1391 |
| PU_male_low | PU_male_medium | -3.4199 | 2.834605 | -1.21 | 0.2280 | -8.9845 | 2.1448 |

**Unclassified *Lactobacillus***

| **PU Gender** | **Estimate** | **Std Error** | **DF** | **Lower 95%** | **Upper 95%** | **Arithmetic Mean Estimate** | **N** |
| --- | --- | --- | --- | --- | --- | --- | --- |
| PU_female_high | 25.129815 | 2.8503812 | 753 | 19.534177 | 30.725454 | 25.129815 | 50 |
| PU_female_low | 20.887317 | 2.8503812 | 753 | 15.291678 | 26.482955 | 20.887317 | 50 |
| PU_female_medium | 25.101431 | 1.2475780 | 753 | 22.652287 | 27.550576 | 25.101431 | 261 |
| PU_male_high | 15.042881 | 2.8503812 | 753 | 9.447243 | 20.638520 | 15.042881 | 50 |
| PU_male_low | 21.999348 | 2.8503812 | 753 | 16.403710 | 27.594987 | 21.999348 | 50 |
| PU_male_medium | 21.416683 | 1.2428253 | 753 | 18.976869 | 23.856497 | 21.416683 | 263 |

| **PU Gender** | **-PU Gender** | **Difference** | **Std Error** | **t Ratio** | **Prob>\|t\|** | **Lower 95%** | **Upper 95%** |
| --- | --- | --- | --- | --- | --- | --- | --- |
| PU_female_high | PU_female_low | 4.2425 | 4.031048 | 1.05 | 0.2929 | -3.6709 | 12.1559 |
| PU_female_high | PU_female_medium | 0.0284 | 3.111450 | 0.01 | 0.9927 | -6.0798 | 6.1365 |
| PU_female_high | PU_male_high | 10.0869 | 4.031048 | 2.50 | 0.0125* | 2.1735 | 18.0004 |
| PU_female_high | PU_male_low | 3.1305 | 4.031048 | 0.78 | 0.4376 | -4.7830 | 11.0439 |
| PU_female_high | PU_male_medium | 3.7131 | 3.109548 | 1.19 | 0.2328 | -2.3913 | 9.8175 |
| PU_female_low | PU_female_medium | -4.2141 | 3.111450 | -1.35 | 0.1760 | -10.3223 | 1.8940 |
| PU_female_low | PU_male_high | 5.8444 | 4.031048 | 1.45 | 0.1475 | -2.0690 | 13.7579 |
| PU_female_low | PU_male_low | -1.1120 | 4.031048 | -0.28 | 0.7827 | -9.0255 | 6.8014 |
| PU_female_low | PU_male_medium | -0.5294 | 3.109548 | -0.17 | 0.8649 | -6.6338 | 5.5750 |
| PU_female_medium | PU_male_high | 10.0586 | 3.111450 | 3.23 | 0.0013* | 3.9504 | 16.1667 |
| PU_female_medium | PU_male_low | 3.1021 | 3.111450 | 1.00 | 0.3191 | -3.0061 | 9.2102 |
| PU_female_medium | PU_male_medium | 3.6847 | 1.760984 | 2.09 | 0.0367* | 0.2277 | 7.1418 |
| PU_male_high | PU_male_low | -6.9565 | 4.031048 | -1.73 | 0.0848 | -14.8699 | 0.9570 |
| PU_male_high | PU_male_medium | -6.3738 | 3.109548 | -2.05 | 0.0407* | -12.4782 | -0.2694 |
| PU_male_low | PU_male_medium | 0.5827 | 3.109548 | 0.19 | 0.8514 | -5.5217 | 6.6871 |

**Unclassified *Clostridium* sensu stricto1**

| **PU Gender** | **Estimate** | **Std Error** | **DF** | **Lower 95%** | **Upper 95%** | **Arithmetic Mean Estimate** | **N** |
| --- | --- | --- | --- | --- | --- | --- | --- |
| PU_female_high | 7.797617 | 2.1370915 | 753 | 3.602252 | 11.992983 | 7.797617 | 50 |
| PU_female_low | 11.851688 | 2.1370915 | 753 | 7.656322 | 16.047054 | 11.851688 | 50 |
| PU_female_medium | 15.896603 | 0.9353796 | 753 | 14.060341 | 17.732865 | 15.896603 | 261 |
| PU_male_high | 10.060631 | 2.1370915 | 753 | 5.865265 | 14.255997 | 10.060631 | 50 |
| PU_male_low | 9.409131 | 2.1370915 | 753 | 5.213765 | 13.604497 | 9.409131 | 50 |
| PU_male_medium | 12.598227 | 0.9318162 | 753 | 10.768960 | 14.427493 | 12.598227 | 263 |

| **PU Gender** | **-PU Gender** | **Difference** | **Std Error** | **t Ratio** | **Prob>\|t\|** | **Lower 95%** | **Upper 95%** |
| --- | --- | --- | --- | --- | --- | --- | --- |
| PU_female_high | PU_female_low | -4.05407 | 3.022304 | -1.34 | 0.1802 | -9.9872 | 1.8791 |
| PU_female_high | PU_female_medium | -8.09899 | 2.332830 | -3.47 | 0.0005* | -12.6786 | -3.5194 |
| PU_female_high | PU_male_high | -2.26301 | 3.022304 | -0.75 | 0.4542 | -8.1962 | 3.6701 |
| PU_female_high | PU_male_low | -1.61151 | 3.022304 | -0.53 | 0.5940 | -7.5447 | 4.3216 |
| PU_female_high | PU_male_medium | -4.80061 | 2.331403 | -2.06 | 0.0398* | -9.3774 | -0.2238 |
| PU_female_low | PU_female_medium | -4.04492 | 2.332830 | -1.73 | 0.0833 | -8.6245 | 0.5347 |
| PU_female_low | PU_male_high | 1.79106 | 3.022304 | 0.59 | 0.5536 | -4.1421 | 7.7242 |
| PU_female_low | PU_male_low | 2.44256 | 3.022304 | 0.81 | 0.4192 | -3.4906 | 8.3757 |
| PU_female_low | PU_male_medium | -0.74654 | 2.331403 | -0.32 | 0.7489 | -5.3234 | 3.8303 |
| PU_female_medium | PU_male_high | 5.83597 | 2.332830 | 2.50 | 0.0126* | 1.2563 | 10.4156 |
| PU_female_medium | PU_male_low | 6.48747 | 2.332830 | 2.78 | 0.0056* | 1.9078 | 11.0671 |
| PU_female_medium | PU_male_medium | 3.29838 | 1.320309 | 2.50 | 0.0127* | 0.7065 | 5.8903 |
| PU_male_high | PU_male_low | 0.65150 | 3.022304 | 0.22 | 0.8294 | -5.2816 | 6.5846 |
| PU_male_high | PU_male_medium | -2.53760 | 2.331403 | -1.09 | 0.2767 | -7.1144 | 2.0392 |
| PU_male_low | PU_male_medium | -3.18910 | 2.331403 | -1.37 | 0.1718 | -7.7659 | 1.3877 |

***Escherichia coli***

| **PU Gender** | **Estimate** | **Std Error** | **DF** | **Lower 95%** | **Upper 95%** | **Arithmetic Mean Estimate** | **N** |
| --- | --- | --- | --- | --- | --- | --- | --- |
| PU_female_high | 6.723986 | 1.3516429 | 753 | 4.0705491 | 9.377422 | 6.723986 | 50 |
| PU_female_low | 12.137077 | 1.3516429 | 753 | 9.4836409 | 14.790514 | 12.137077 | 50 |
| PU_female_medium | 10.959678 | 0.5915980 | 753 | 9.7983004 | 12.121056 | 10.959678 | 261 |
| PU_male_high | 10.967929 | 1.3516429 | 753 | 8.3144924 | 13.621365 | 10.967929 | 50 |
| PU_male_low | 9.824519 | 1.3516429 | 753 | 7.1710828 | 12.477956 | 9.824519 | 50 |
| PU_male_medium | 10.886050 | 0.5893443 | 753 | 9.7290971 | 12.043004 | 10.886050 | 263 |

| **PU Gender** | **-PU Gender** | **Difference** | **Std Error** | **t Ratio** | **Prob>\|t\|** | **Lower 95%** | **Upper 95%** |
| --- | --- | --- | --- | --- | --- | --- | --- |
| PU_female_high | PU_female_low | -5.41309 | 1.911512 | -2.83 | 0.0048* | -9.16562 | -1.66057 |
| PU_female_high | PU_female_medium | -4.23569 | 1.475441 | -2.87 | 0.0042* | -7.13216 | -1.33923 |
| PU_female_high | PU_male_high | -4.24394 | 1.911512 | -2.22 | 0.0267* | -7.99647 | -0.49142 |
| PU_female_high | PU_male_low | -3.10053 | 1.911512 | -1.62 | 0.1052 | -6.85306 | 0.65199 |
| PU_female_high | PU_male_medium | -4.16206 | 1.474539 | -2.82 | 0.0049* | -7.05676 | -1.26737 |
| PU_female_low | PU_female_medium | 1.17740 | 1.475441 | 0.80 | 0.4251 | -1.71907 | 4.07387 |
| PU_female_low | PU_male_high | 1.16915 | 1.911512 | 0.61 | 0.5410 | -2.58338 | 4.92167 |
| PU_female_low | PU_male_low | 2.31256 | 1.911512 | 1.21 | 0.2267 | -1.43997 | 6.06508 |
| PU_female_low | PU_male_medium | 1.25103 | 1.474539 | 0.85 | 0.3965 | -1.64367 | 4.14572 |
| PU_female_medium | PU_male_high | -0.00825 | 1.475441 | -0.01 | 0.9955 | -2.90472 | 2.88822 |
| PU_female_medium | PU_male_low | 1.13516 | 1.475441 | 0.77 | 0.4419 | -1.76131 | 4.03163 |
| PU_female_medium | PU_male_medium | 0.07363 | 0.835054 | 0.09 | 0.9298 | -1.56568 | 1.71294 |
| PU_male_high | PU_male_low | 1.14341 | 1.911512 | 0.60 | 0.5499 | -2.60912 | 4.89594 |
| PU_male_high | PU_male_medium | 0.08188 | 1.474539 | 0.06 | 0.9557 | -2.81282 | 2.97657 |
| PU_male_low | PU_male_medium | -1.06153 | 1.474539 | -0.72 | 0.4718 | -3.95623 | 1.83317 |

***Streptococcus alactolyticus***

| **PU Gender** | **Estimate** | **Std Error** | | **DF** | | **Lower 95%** | **Upper 95%** | **Arithmetic Mean Estimate** | **N** |
| --- | --- | --- | --- | --- | --- | --- | --- | --- | --- |
| PU_female_high | 7.045891 | 2.0804632 | | 753 | | 2.9616928 | 11.130088 | 7.045891 | 50 |
| PU_female_low | 8.537856 | 2.0804632 | | 753 | | 4.4536581 | 12.622054 | 8.537856 | 50 |
| PU_female_medium | 6.234464 | 0.9105940 | | 753 | | 4.4468588 | 8.022068 | 6.234464 | 261 |
| PU_male_high | 8.002447 | 2.0804632 | | 753 | | 3.9182496 | 12.086645 | 8.002447 | 50 |
| PU_male_low | 12.931992 | 2.0804632 | | 753 | | 8.8477944 | 17.016190 | 12.931992 | 50 |
| PU_male_medium | 8.211126 | 0.9071251 | | 753 | | 6.4303307 | 9.991920 | 8.211126 | 263 |
|  |  |  | |  | |  |  |  |  |
| **PU Gender** | **-PU Gender** | | **Difference** | | **Std Error** | **t Ratio** | **Prob>\|t\|** | **Lower 95%** | **Upper 95%** |
| PU_female_high | PU_female_low | | -1.49197 | | 2.942219 | -0.51 | 0.6122 | -7.2679 | 4.28396 |
| PU_female_high | PU_female_medium | | 0.81143 | | 2.271015 | 0.36 | 0.7210 | -3.6468 | 5.26970 |
| PU_female_high | PU_male_high | | -0.95656 | | 2.942219 | -0.33 | 0.7452 | -6.7325 | 4.81937 |
| PU_female_high | PU_male_low | | -5.88610 | | 2.942219 | -2.00 | 0.0458* | -11.6620 | -0.11017 |
| PU_female_high | PU_male_medium | | -1.16523 | | 2.269626 | -0.51 | 0.6078 | -5.6208 | 3.29031 |
| PU_female_low | PU_female_medium | | 2.30339 | | 2.271015 | 1.01 | 0.3108 | -2.1549 | 6.76167 |
| PU_female_low | PU_male_high | | 0.53541 | | 2.942219 | 0.18 | 0.8557 | -5.2405 | 6.31134 |
| PU_female_low | PU_male_low | | -4.39414 | | 2.942219 | -1.49 | 0.1357 | -10.1701 | 1.38179 |
| PU_female_low | PU_male_medium | | 0.32673 | | 2.269626 | 0.14 | 0.8856 | -4.1288 | 4.78228 |
| PU_female_medium | PU_male_high | | -1.76798 | | 2.271015 | -0.78 | 0.4365 | -6.2263 | 2.69029 |
| PU_female_medium | PU_male_low | | -6.69753 | | 2.271015 | -2.95 | 0.0033* | -11.1558 | -2.23926 |
| PU_female_medium | PU_male_medium | | -1.97666 | | 1.285324 | -1.54 | 0.1245 | -4.4999 | 0.54658 |
| PU_male_high | PU_male_low | | -4.92954 | | 2.942219 | -1.68 | 0.0943 | -10.7055 | 0.84638 |
| PU_male_high | PU_male_medium | | -0.20868 | | 2.269626 | -0.09 | 0.9268 | -4.6642 | 4.24687 |
| PU_male_low | PU_male_medium | | 4.72087 | | 2.269626 | 2.08 | 0.0379* | 0.2653 | 9.17641 |

***Enterococcus faecium***

| **PU Gender** | **Estimate** | **Std Error** | **DF** | **Lower 95%** | **Upper 95%** | **Arithmetic Mean Estimate** | **N** |
| --- | --- | --- | --- | --- | --- | --- | --- |
| PU_female_high | 0.87315828 | 0.48805053 | 753 | -0.0849432 | 1.8312597 | 0.87315828 | 50 |
| PU_female_low | 0.90883386 | 0.48805053 | 753 | -0.0492676 | 1.8669353 | 0.90883386 | 50 |
| PU_female_medium | 0.96652190 | 0.21361391 | 753 | 0.5471723 | 1.3858715 | 0.96652190 | 261 |
| PU_male_high | 0.66023534 | 0.48805053 | 753 | -0.2978661 | 1.6183368 | 0.66023534 | 50 |
| PU_male_low | 0.66618125 | 0.48805053 | 753 | -0.2919202 | 1.6242827 | 0.66618125 | 50 |
| PU_male_medium | 0.63829015 | 0.21280014 | 753 | 0.2205381 | 1.0560422 | 0.63829015 | 263 |

| **PU Gender** | **-PU Gender** | **Difference** | **Std Error** | **t Ratio** | **Prob>\|t\|** | **Lower 95%** | **Upper 95%** |
| --- | --- | --- | --- | --- | --- | --- | --- |
| PU_female_high | PU_female_low | -0.035676 | 0.6902077 | -0.05 | 0.9588 | -1.39064 | 1.319285 |
| PU_female_high | PU_female_medium | -0.093364 | 0.5327516 | -0.18 | 0.8609 | -1.13922 | 0.952491 |
| PU_female_high | PU_male_high | 0.212923 | 0.6902077 | 0.31 | 0.7578 | -1.14204 | 1.567883 |
| PU_female_high | PU_male_low | 0.206977 | 0.6902077 | 0.30 | 0.7644 | -1.14798 | 1.561937 |
| PU_female_high | PU_male_medium | 0.234868 | 0.5324258 | 0.44 | 0.6592 | -0.81035 | 1.280084 |
| PU_female_low | PU_female_medium | -0.057688 | 0.5327516 | -0.11 | 0.9138 | -1.10354 | 0.988167 |
| PU_female_low | PU_male_high | 0.248599 | 0.6902077 | 0.36 | 0.7188 | -1.10636 | 1.603559 |
| PU_female_low | PU_male_low | 0.242653 | 0.6902077 | 0.35 | 0.7253 | -1.11231 | 1.597613 |
| PU_female_low | PU_male_medium | 0.270544 | 0.5324258 | 0.51 | 0.6115 | -0.77467 | 1.315759 |
| PU_female_medium | PU_male_high | 0.306287 | 0.5327516 | 0.57 | 0.5655 | -0.73957 | 1.352141 |
| PU_female_medium | PU_male_low | 0.300341 | 0.5327516 | 0.56 | 0.5731 | -0.74551 | 1.346196 |
| PU_female_medium | PU_male_medium | 0.328232 | 0.3015208 | 1.09 | 0.2767 | -0.26369 | 0.920153 |
| PU_male_high | PU_male_low | -0.005946 | 0.6902077 | -0.01 | 0.9931 | -1.36091 | 1.349014 |
| PU_male_high | PU_male_medium | 0.021945 | 0.5324258 | 0.04 | 0.9671 | -1.02327 | 1.067161 |
| PU_male_low | PU_male_medium | 0.027891 | 0.5324258 | 0.05 | 0.9582 | -1.01732 | 1.073106 |
|  |  |  |  |  |  |  |  |
|  |  |  |  |  |  |  |  |
|  |  |  |  |  |  |  |  |
|  |  |  |  |  |  |  |  |
|  |  |  |  |  |  |  |  |
|  |  |  |  |  |  |  |  |

1. **Calcium utilization**

**Unclassified Clostridiaceae1**

| **CaU Gender** | **Estimate** | **Std Error** | **DF** | **Lower 95%** | **Upper 95%** | **Arithmetic Mean Estimate** | **N** |
| --- | --- | --- | --- | --- | --- | --- | --- |
| CaU_female_high | 32.489594 | 2.6223129 | 753 | 27.341681 | 37.637507 | 32.489594 | 50 |
| CaU_female_low | 25.387795 | 2.6223129 | 753 | 20.239881 | 30.535708 | 25.387795 | 50 |
| CaU_female_medium | 28.572073 | 1.1477552 | 753 | 26.318892 | 30.825253 | 28.572073 | 261 |
| CaU_male_high | 37.474924 | 2.6223129 | 753 | 32.327011 | 42.622837 | 37.474924 | 50 |
| CaU_male_low | 28.417672 | 2.6223129 | 753 | 23.269759 | 33.565585 | 28.417672 | 50 |
| CaU_male_medium | 29.566390 | 1.1433827 | 753 | 27.321793 | 31.810987 | 29.566390 | 263 |

| **CaU Gender** | **-CaU Gender** | **Difference** | **Std Error** | **t Ratio** | **Prob>\|t\|** | **Lower 95%** | **Upper 95%** |
| --- | --- | --- | --- | --- | --- | --- | --- |
| CaU_female_high | CaU_female_low | 7.1018 | 3.708510 | 1.92 | 0.0559 | -0.1784 | 14.3820 |
| CaU_female_high | CaU_female_medium | 3.9175 | 2.862493 | 1.37 | 0.1715 | -1.7019 | 9.5369 |
| CaU_female_high | CaU_male_high | -4.9853 | 3.708510 | -1.34 | 0.1793 | -12.2656 | 2.2949 |
| CaU_female_high | CaU_male_low | 4.0719 | 3.708510 | 1.10 | 0.2726 | -3.2083 | 11.3522 |
| CaU_female_high | CaU_male_medium | 2.9232 | 2.860743 | 1.02 | 0.3072 | -2.6928 | 8.5392 |
| CaU_female_low | CaU_female_medium | -3.1843 | 2.862493 | -1.11 | 0.2663 | -8.8037 | 2.4351 |
| CaU_female_low | CaU_male_high | -12.0871 | 3.708510 | -3.26 | 0.0012* | -19.3674 | -4.8069 |
| CaU_female_low | CaU_male_low | -3.0299 | 3.708510 | -0.82 | 0.4142 | -10.3101 | 4.2504 |
| CaU_female_low | CaU_male_medium | -4.1786 | 2.860743 | -1.46 | 0.1445 | -9.7946 | 1.4374 |
| CaU_female_medium | CaU_male_high | -8.9029 | 2.862493 | -3.11 | 0.0019* | -14.5223 | -3.2834 |
| CaU_female_medium | CaU_male_low | 0.1544 | 2.862493 | 0.05 | 0.9570 | -5.4650 | 5.7738 |
| CaU_female_medium | CaU_male_medium | -0.9943 | 1.620082 | -0.61 | 0.5396 | -4.1747 | 2.1861 |
| CaU_male_high | CaU_male_low | 9.0573 | 3.708510 | 2.44 | 0.0148* | 1.7770 | 16.3375 |
| CaU_male_high | CaU_male_medium | 7.9085 | 2.860743 | 2.76 | 0.0058* | 2.2926 | 13.5245 |
| CaU_male_low | CaU_male_medium | -1.1487 | 2.860743 | -0.40 | 0.6881 | -6.7647 | 4.4673 |

**Unclassified *Lactobacillus***

| **CaU Gender** | **Estimate** | **Std Error** | **DF** | **Lower 95%** | **Upper 95%** | **Arithmetic Mean Estimate** | **N** |
| --- | --- | --- | --- | --- | --- | --- | --- |
| CaU_female_high | 25.108605 | 2.8561456 | 753 | 19.501650 | 30.715560 | 25.108605 | 50 |
| CaU_female_low | 24.313484 | 2.8561456 | 753 | 18.706529 | 29.920439 | 24.313484 | 50 |
| CaU_female_medium | 24.449141 | 1.2501010 | 753 | 21.995043 | 26.903238 | 24.449141 | 261 |
| CaU_male_high | 16.320415 | 2.8561456 | 753 | 10.713461 | 21.927370 | 16.320415 | 50 |
| CaU_male_low | 23.368407 | 2.8561456 | 753 | 17.761452 | 28.975362 | 23.368407 | 50 |
| CaU_male_medium | 20.913529 | 1.2453387 | 753 | 18.468780 | 23.358277 | 20.913529 | 263 |

| **CaU Gender** | **-CaU Gender** | **Difference** | **Std Error** | **t Ratio** | **Prob>\|t\|** | **Lower 95%** | **Upper 95%** |
| --- | --- | --- | --- | --- | --- | --- | --- |
| CaU_female_high | CaU_female_low | 0.7951 | 4.039200 | 0.20 | 0.8440 | -7.1343 | 8.7246 |
| CaU_female_high | CaU_female_medium | 0.6595 | 3.117743 | 0.21 | 0.8325 | -5.4610 | 6.7800 |
| CaU_female_high | CaU_male_high | 8.7882 | 4.039200 | 2.18 | 0.0299* | 0.8588 | 16.7176 |
| CaU_female_high | CaU_male_low | 1.7402 | 4.039200 | 0.43 | 0.6667 | -6.1892 | 9.6696 |
| CaU_female_high | CaU_male_medium | 4.1951 | 3.115836 | 1.35 | 0.1786 | -1.9217 | 10.3118 |
| CaU_female_low | CaU_female_medium | -0.1357 | 3.117743 | -0.04 | 0.9653 | -6.2562 | 5.9848 |
| CaU_female_low | CaU_male_high | 7.9931 | 4.039200 | 1.98 | 0.0482* | 0.0636 | 15.9225 |
| CaU_female_low | CaU_male_low | 0.9451 | 4.039200 | 0.23 | 0.8151 | -6.9844 | 8.8745 |
| CaU_female_low | CaU_male_medium | 3.4000 | 3.115836 | 1.09 | 0.2755 | -2.7168 | 9.5167 |
| CaU_female_medium | CaU_male_high | 8.1287 | 3.117743 | 2.61 | 0.0093* | 2.0082 | 14.2492 |
| CaU_female_medium | CaU_male_low | 1.0807 | 3.117743 | 0.35 | 0.7290 | -5.0398 | 7.2012 |
| CaU_female_medium | CaU_male_medium | 3.5356 | 1.764546 | 2.00 | 0.0455* | 0.0716 | 6.9996 |
| CaU_male_high | CaU_male_low | -7.0480 | 4.039200 | -1.74 | 0.0814 | -14.9774 | 0.8814 |
| CaU_male_high | CaU_male_medium | -4.5931 | 3.115836 | -1.47 | 0.1409 | -10.7099 | 1.5236 |
| CaU_male_low | CaU_male_medium | 2.4549 | 3.115836 | 0.79 | 0.4310 | -3.6619 | 8.5716 |

**Unclassified *Clostridium* sensu stricto 1**

| **CaU Gender** | **Estimate** | **Std Error** | **DF** | **Lower 95%** | **Upper 95%** | **Arithmetic Mean Estimate** | **N** |
| --- | --- | --- | --- | --- | --- | --- | --- |
| CaU_female_high | 9.058401 | 2.1496329 | 753 | 4.838415 | 13.278387 | 9.058401 | 50 |
| CaU_female_low | 14.274486 | 2.1496329 | 753 | 10.054500 | 18.494472 | 14.274486 | 50 |
| CaU_female_medium | 15.190936 | 0.9408688 | 753 | 13.343899 | 17.037974 | 15.190936 | 261 |
| CaU_male_high | 10.990355 | 2.1496329 | 753 | 6.770369 | 15.210341 | 10.990355 | 50 |
| CaU_male_low | 11.122698 | 2.1496329 | 753 | 6.902712 | 15.342684 | 11.122698 | 50 |
| CaU_male_medium | 12.095700 | 0.9372845 | 753 | 10.255699 | 13.935701 | 12.095700 | 263 |

| **CaU Gender** | **-CaU Gender** | **Difference** | **Std Error** | **t Ratio** | **Prob>\|t\|** | **Lower 95%** | **Upper 95%** |
| --- | --- | --- | --- | --- | --- | --- | --- |
| CaU_female_high | CaU_female_low | -5.21609 | 3.040040 | -1.72 | 0.0866 | -11.1840 | 0.75188 |
| CaU_female_high | CaU_female_medium | -6.13254 | 2.346520 | -2.61 | 0.0091* | -10.7390 | -1.52604 |
| CaU_female_high | CaU_male_high | -1.93195 | 3.040040 | -0.64 | 0.5253 | -7.8999 | 4.03601 |
| CaU_female_high | CaU_male_low | -2.06430 | 3.040040 | -0.68 | 0.4973 | -8.0323 | 3.90366 |
| CaU_female_high | CaU_male_medium | -3.03730 | 2.345085 | -1.30 | 0.1957 | -7.6410 | 1.56638 |
| CaU_female_low | CaU_female_medium | -0.91645 | 2.346520 | -0.39 | 0.6962 | -5.5229 | 3.69005 |
| CaU_female_low | CaU_male_high | 3.28413 | 3.040040 | 1.08 | 0.2804 | -2.6838 | 9.25209 |
| CaU_female_low | CaU_male_low | 3.15179 | 3.040040 | 1.04 | 0.3002 | -2.8162 | 9.11975 |
| CaU_female_low | CaU_male_medium | 2.17879 | 2.345085 | 0.93 | 0.3531 | -2.4249 | 6.78247 |
| CaU_female_medium | CaU_male_high | 4.20058 | 2.346520 | 1.79 | 0.0738 | -0.4059 | 8.80708 |
| CaU_female_medium | CaU_male_low | 4.06824 | 2.346520 | 1.73 | 0.0834 | -0.5383 | 8.67474 |
| CaU_female_medium | CaU_male_medium | 3.09524 | 1.328057 | 2.33 | 0.0200* | 0.4881 | 5.70237 |
| CaU_male_high | CaU_male_low | -0.13234 | 3.040040 | -0.04 | 0.9653 | -6.1003 | 5.83562 |
| CaU_male_high | CaU_male_medium | -1.10534 | 2.345085 | -0.47 | 0.6375 | -5.7090 | 3.49834 |
| CaU_male_low | CaU_male_medium | -0.97300 | 2.345085 | -0.41 | 0.6783 | -5.5767 | 3.63068 |

***Escherichia coli***

| **CaU Gender** | **Estimate** | **Std Error** | **DF** | **Lower 95%** | **Upper 95%** | **Arithmetic Mean Estimate** | **N** |
| --- | --- | --- | --- | --- | --- | --- | --- |
| CaU_female_high | 6.910912 | 1.3506962 | 753 | 4.259334 | 9.562490 | 6.910912 | 50 |
| CaU_female_low | 13.151423 | 1.3506962 | 753 | 10.499845 | 15.803001 | 13.151423 | 50 |
| CaU_female_medium | 10.729549 | 0.5911837 | 753 | 9.568985 | 11.890113 | 10.729549 | 261 |
| CaU_male_high | 10.474913 | 1.3506962 | 753 | 7.823335 | 13.126491 | 10.474913 | 50 |
| CaU_male_low | 11.558874 | 1.3506962 | 753 | 8.907296 | 14.210452 | 11.558874 | 50 |
| CaU_male_medium | 10.650054 | 0.5889315 | 753 | 9.493911 | 11.806197 | 10.650054 | 263 |

| **CaU Gender** | **-CaU Gender** | **Difference** | **Std Error** | **t Ratio** | **Prob>\|t\|** | **Lower 95%** | **Upper 95%** |
| --- | --- | --- | --- | --- | --- | --- | --- |
| CaU_female_high | CaU_female_low | -6.24051 | 1.910173 | -3.27 | 0.0011* | -9.99041 | -2.49061 |
| CaU_female_high | CaU_female_medium | -3.81864 | 1.474408 | -2.59 | 0.0098* | -6.71308 | -0.92420 |
| CaU_female_high | CaU_male_high | -3.56400 | 1.910173 | -1.87 | 0.0625 | -7.31390 | 0.18590 |
| CaU_female_high | CaU_male_low | -4.64796 | 1.910173 | -2.43 | 0.0152* | -8.39786 | -0.89806 |
| CaU_female_high | CaU_male_medium | -3.73914 | 1.473506 | -2.54 | 0.0114* | -6.63181 | -0.84647 |
| CaU_female_low | CaU_female_medium | 2.42187 | 1.474408 | 1.64 | 0.1009 | -0.47257 | 5.31631 |
| CaU_female_low | CaU_male_high | 2.67651 | 1.910173 | 1.40 | 0.1616 | -1.07339 | 6.42641 |
| CaU_female_low | CaU_male_low | 1.59255 | 1.910173 | 0.83 | 0.4047 | -2.15735 | 5.34245 |
| CaU_female_low | CaU_male_medium | 2.50137 | 1.473506 | 1.70 | 0.0900 | -0.39130 | 5.39404 |
| CaU_female_medium | CaU_male_high | 0.25464 | 1.474408 | 0.17 | 0.8629 | -2.63980 | 3.14907 |
| CaU_female_medium | CaU_male_low | -0.82932 | 1.474408 | -0.56 | 0.5740 | -3.72376 | 2.06511 |
| CaU_female_medium | CaU_male_medium | 0.07949 | 0.834469 | 0.10 | 0.9241 | -1.55867 | 1.71766 |
| CaU_male_high | CaU_male_low | -1.08396 | 1.910173 | -0.57 | 0.5706 | -4.83386 | 2.66594 |
| CaU_male_high | CaU_male_medium | -0.17514 | 1.473506 | -0.12 | 0.9054 | -3.06781 | 2.71753 |
| CaU_male_low | CaU_male_medium | 0.90882 | 1.473506 | 0.62 | 0.5376 | -1.98385 | 3.80149 |

***Streptococcus alactolyticus***

| **CaU Gender** | **Estimate** | **Std Error** | **DF** | **Lower 95%** | **Upper 95%** | **Arithmetic Mean Estimate** | **N** |
| --- | --- | --- | --- | --- | --- | --- | --- |
| CaU_female_high | 10.208999 | 2.0801208 | 753 | 6.1254739 | 14.292525 | 10.208999 | 50 |
| CaU_female_low | 4.991869 | 2.0801208 | 753 | 0.9083441 | 9.075395 | 4.991869 | 50 |
| CaU_female_medium | 6.307812 | 0.9104441 | 753 | 4.5205013 | 8.095122 | 6.307812 | 261 |
| CaU_male_high | 6.425196 | 2.0801208 | 753 | 2.3416706 | 10.508721 | 6.425196 | 50 |
| CaU_male_low | 7.775978 | 2.0801208 | 753 | 3.6924523 | 11.859503 | 7.775978 | 50 |
| CaU_male_medium | 9.491214 | 0.9069757 | 753 | 7.7107124 | 11.271716 | 9.491214 | 263 |

| **CaU Gender** | **-CaU Gender** | **Difference** | **Std Error** | **t Ratio** | **Prob>\|t\|** | **Lower 95%** | **Upper 95%** |
| --- | --- | --- | --- | --- | --- | --- | --- |
| CaU_female_high | CaU_female_low | 5.21713 | 2.941735 | 1.77 | 0.0766 | -0.55785 | 10.9921 |
| CaU_female_high | CaU_female_medium | 3.90119 | 2.270641 | 1.72 | 0.0862 | -0.55635 | 8.3587 |
| CaU_female_high | CaU_male_high | 3.78380 | 2.941735 | 1.29 | 0.1988 | -1.99117 | 9.5588 |
| CaU_female_high | CaU_male_low | 2.43302 | 2.941735 | 0.83 | 0.4085 | -3.34196 | 8.2080 |
| CaU_female_high | CaU_male_medium | 0.71779 | 2.269253 | 0.32 | 0.7519 | -3.73703 | 5.1726 |
| CaU_female_low | CaU_female_medium | -1.31594 | 2.270641 | -0.58 | 0.5624 | -5.77348 | 3.1416 |
| CaU_female_low | CaU_male_high | -1.43333 | 2.941735 | -0.49 | 0.6262 | -7.20830 | 4.3417 |
| CaU_female_low | CaU_male_low | -2.78411 | 2.941735 | -0.95 | 0.3442 | -8.55909 | 2.9909 |
| CaU_female_low | CaU_male_medium | -4.49934 | 2.269253 | -1.98 | 0.0478* | -8.95416 | -0.0445 |
| CaU_female_medium | CaU_male_high | -0.11738 | 2.270641 | -0.05 | 0.9588 | -4.57492 | 4.3402 |
| CaU_female_medium | CaU_male_low | -1.46817 | 2.270641 | -0.65 | 0.5181 | -5.92571 | 2.9894 |
| CaU_female_medium | CaU_male_medium | -3.18340 | 1.285112 | -2.48 | 0.0135* | -5.70623 | -0.6606 |
| CaU_male_high | CaU_male_low | -1.35078 | 2.941735 | -0.46 | 0.6462 | -7.12576 | 4.4242 |
| CaU_male_high | CaU_male_medium | -3.06602 | 2.269253 | -1.35 | 0.1771 | -7.52083 | 1.3888 |
| CaU_male_low | CaU_male_medium | -1.71524 | 2.269253 | -0.76 | 0.4500 | -6.17005 | 2.7396 |

***Enterococcus faecium***

| **CaU Gender** | **Estimate** | **Std Error** | **DF** | **Lower 95%** | **Upper 95%** | **Arithmetic Mean Estimate** | **N** |
| --- | --- | --- | --- | --- | --- | --- | --- |
| CaU_female_high | 1.0514501 | 0.76519097 | 753 | -0.450711 | 2.5536113 | 1.0514501 | 50 |
| CaU_female_low | 2.3583525 | 0.76519097 | 753 | 0.856191 | 3.8605137 | 2.3583525 | 50 |
| CaU_female_medium | 1.1321759 | 0.33491499 | 753 | 0.474698 | 1.7896540 | 1.1321759 | 261 |
| CaU_male_high | 2.1941066 | 0.76519097 | 753 | 0.691945 | 3.6962679 | 2.1941066 | 50 |
| CaU_male_low | 1.1413537 | 0.76519097 | 753 | -0.360808 | 2.6435149 | 1.1413537 | 50 |
| CaU_male_medium | 1.8026372 | 0.33363912 | 753 | 1.147664 | 2.4576106 | 1.8026372 | 263 |

| **CaU Gender** | **-CaU Gender** | **Difference** | **Std Error** | **t Ratio** | **Prob>\|t\|** | **Lower 95%** | **Upper 95%** |
| --- | --- | --- | --- | --- | --- | --- | --- |
| CaU_female_high | CaU_female_low | -1.30690 | 1.082143 | -1.21 | 0.2275 | -3.43128 | 0.817474 |
| CaU_female_high | CaU_female_medium | -0.08073 | 0.835276 | -0.10 | 0.9230 | -1.72047 | 1.559020 |
| CaU_female_high | CaU_male_high | -1.14266 | 1.082143 | -1.06 | 0.2913 | -3.26703 | 0.981720 |
| CaU_female_high | CaU_male_low | -0.08990 | 1.082143 | -0.08 | 0.9338 | -2.21428 | 2.034473 |
| CaU_female_high | CaU_male_medium | -0.75119 | 0.834765 | -0.90 | 0.3685 | -2.38993 | 0.887556 |
| CaU_female_low | CaU_female_medium | 1.22618 | 0.835276 | 1.47 | 0.1425 | -0.41357 | 2.865922 |
| CaU_female_low | CaU_male_high | 0.16425 | 1.082143 | 0.15 | 0.8794 | -1.96013 | 2.288623 |
| CaU_female_low | CaU_male_low | 1.21700 | 1.082143 | 1.12 | 0.2611 | -0.90738 | 3.341376 |
| CaU_female_low | CaU_male_medium | 0.55572 | 0.834765 | 0.67 | 0.5058 | -1.08303 | 2.194458 |
| CaU_female_medium | CaU_male_high | -1.06193 | 0.835276 | -1.27 | 0.2040 | -2.70168 | 0.577815 |
| CaU_female_medium | CaU_male_low | -0.00918 | 0.835276 | -0.01 | 0.9912 | -1.64892 | 1.630568 |
| CaU_female_medium | CaU_male_medium | -0.67046 | 0.472740 | -1.42 | 0.1565 | -1.59851 | 0.257584 |
| CaU_male_high | CaU_male_low | 1.05275 | 1.082143 | 0.97 | 0.3309 | -1.07162 | 3.177130 |
| CaU_male_high | CaU_male_medium | 0.39147 | 0.834765 | 0.47 | 0.6392 | -1.24727 | 2.030212 |
| CaU_male_low | CaU_male_medium | -0.66128 | 0.834765 | -0.79 | 0.4285 | -2.30003 | 0.977459 |

**C- Feed intake**

**Unclassified Clostridiaceae1**

| **Feed intake Gender** | **Estimate** | **Std Error** | **DF** | **Lower 95%** | **Upper 95%** | **Arithmetic Mean Estimate** | **N** |
| --- | --- | --- | --- | --- | --- | --- | --- |
| FI_female_high | 40.894289 | 2.4762081 | 753 | 36.033197 | 45.755382 | 40.894289 | 50 |
| FI_female_low | 24.278384 | 2.4762081 | 753 | 19.417292 | 29.139476 | 24.278384 | 50 |
| FI_female_medium | 27.174509 | 1.0838069 | 753 | 25.046866 | 29.302151 | 27.174509 | 261 |
| FI_male_high | 49.159110 | 2.4762081 | 753 | 44.298017 | 54.020202 | 49.159110 | 50 |
| FI_male_low | 20.438314 | 2.4762081 | 753 | 15.577221 | 25.299406 | 20.438314 | 50 |
| FI_male_medium | 28.862051 | 1.0796781 | 753 | 26.742514 | 30.981587 | 28.862051 | 263 |

| **Feed intake Gender** | **-Feed intake Gender** | **Difference** | **Std Error** | **t Ratio** | **Prob>\|t\|** | **Lower 95%** | **Upper 95%** |
| --- | --- | --- | --- | --- | --- | --- | --- |
| FI_female_high | FI_female_low | 16.6159 | 3.501887 | 4.74 | <.0001* | 9.7413 | 23.4905 |
| FI_female_high | FI_female_medium | 13.7198 | 2.703006 | 5.08 | <.0001* | 8.4135 | 19.0261 |
| FI_female_high | FI_male_high | -8.2648 | 3.501887 | -2.36 | 0.0185* | -15.1394 | -1.3902 |
| FI_female_high | FI_male_low | 20.4560 | 3.501887 | 5.84 | <.0001* | 13.5814 | 27.3306 |
| FI_female_high | FI_male_medium | 12.0322 | 2.701354 | 4.45 | <.0001* | 6.7292 | 17.3353 |
| FI_female_low | FI_female_medium | -2.8961 | 2.703006 | -1.07 | 0.2843 | -8.2024 | 2.4102 |
| FI_female_low | FI_male_high | -24.8807 | 3.501887 | -7.10 | <.0001* | -31.7553 | -18.0061 |
| FI_female_low | FI_male_low | 3.8401 | 3.501887 | 1.10 | 0.2732 | -3.0346 | 10.7147 |
| FI_female_low | FI_male_medium | -4.5837 | 2.701354 | -1.70 | 0.0901 | -9.8867 | 0.7194 |
| FI_female_medium | FI_male_high | -21.9846 | 2.703006 | -8.13 | <.0001* | -27.2909 | -16.6783 |
| FI_female_medium | FI_male_low | 6.7362 | 2.703006 | 2.49 | 0.0129* | 1.4299 | 12.0425 |
| FI_female_medium | FI_male_medium | -1.6875 | 1.529818 | -1.10 | 0.2703 | -4.6908 | 1.3157 |
| FI_male_high | FI_male_low | 28.7208 | 3.501887 | 8.20 | <.0001* | 21.8462 | 35.5954 |
| FI_male_high | FI_male_medium | 20.2971 | 2.701354 | 7.51 | <.0001* | 14.9940 | 25.6001 |
| FI_male_low | FI_male_medium | -8.4237 | 2.701354 | -3.12 | 0.0019* | -13.7268 | -3.1207 |

**Unclassified *Lactobacillus***

| **Feed intake Gender** | **Estimate** | **Std Error** | **DF** | **Lower 95%** | **Upper 95%** | **Arithmetic Mean Estimate** | **N** |
| --- | --- | --- | --- | --- | --- | --- | --- |
| FI_female_high | 14.440172 | 2.8123477 | 753 | 8.919197 | 19.961146 | 14.440172 | 50 |
| FI_female_low | 23.726048 | 2.8123477 | 753 | 18.205073 | 29.247022 | 23.726048 | 50 |
| FI_female_medium | 26.605438 | 1.2309312 | 753 | 24.188973 | 29.021902 | 26.605438 | 261 |
| FI_male_high | 12.927782 | 2.8123477 | 753 | 7.406808 | 18.448757 | 12.927782 | 50 |
| FI_male_low | 25.979150 | 2.8123477 | 753 | 20.458176 | 31.500124 | 25.979150 | 50 |
| FI_male_medium | 21.062177 | 1.2262419 | 753 | 18.654918 | 23.469436 | 21.062177 | 263 |

| **Feed intake Gender** | **-Feed intake Gender** | **Difference** | **Std Error** | **t Ratio** | **Prob>\|t\|** | **Lower 95%** | **Upper 95%** |
| --- | --- | --- | --- | --- | --- | --- | --- |
| FI_female_high | FI_female_low | -9.2859 | 3.977260 | -2.33 | 0.0198* | -17.0937 | -1.4780 |
| FI_female_high | FI_female_medium | -12.1653 | 3.069933 | -3.96 | <.0001* | -18.1919 | -6.1386 |
| FI_female_high | FI_male_high | 1.5124 | 3.977260 | 0.38 | 0.7039 | -6.2954 | 9.3202 |
| FI_female_high | FI_male_low | -11.5390 | 3.977260 | -2.90 | 0.0038* | -19.3468 | -3.7311 |
| FI_female_high | FI_male_medium | -6.6220 | 3.068056 | -2.16 | 0.0312* | -12.6450 | -0.5990 |
| FI_female_low | FI_female_medium | -2.8794 | 3.069933 | -0.94 | 0.3486 | -8.9060 | 3.1473 |
| FI_female_low | FI_male_high | 10.7983 | 3.977260 | 2.72 | 0.0068* | 2.9904 | 18.6061 |
| FI_female_low | FI_male_low | -2.2531 | 3.977260 | -0.57 | 0.5712 | -10.0609 | 5.5547 |
| FI_female_low | FI_male_medium | 2.6639 | 3.068056 | 0.87 | 0.3855 | -3.3591 | 8.6868 |
| FI_female_medium | FI_male_high | 13.6777 | 3.069933 | 4.46 | <.0001* | 7.6510 | 19.7043 |
| FI_female_medium | FI_male_low | 0.6263 | 3.069933 | 0.20 | 0.8384 | -5.4004 | 6.6529 |
| FI_female_medium | FI_male_medium | 5.5433 | 1.737487 | 3.19 | 0.0015* | 2.1324 | 8.9542 |
| FI_male_high | FI_male_low | -13.0514 | 3.977260 | -3.28 | 0.0011* | -20.8592 | -5.2435 |
| FI_male_high | FI_male_medium | -8.1344 | 3.068056 | -2.65 | 0.0082* | -14.1574 | -2.1114 |
| FI_male_low | FI_male_medium | 4.9170 | 3.068056 | 1.60 | 0.1094 | -1.1060 | 10.9399 |

**Unclassified *Clostridium* sensu stricto 1**

| **Feed intake Gender** | **Estimate** | **Std Error** | **DF** | **Lower 95%** | **Upper 95%** | **Arithmetic Mean Estimate** | **N** |
| --- | --- | --- | --- | --- | --- | --- | --- |
| FI_female_high | 13.881817 | 2.1547856 | 753 | 9.651716 | 18.111918 | 13.881817 | 50 |
| FI_female_low | 13.180275 | 2.1547856 | 753 | 8.950173 | 17.410376 | 13.180275 | 50 |
| FI_female_medium | 14.476529 | 0.9431240 | 753 | 12.625064 | 16.327994 | 14.476529 | 261 |
| FI_male_high | 8.285485 | 2.1547856 | 753 | 4.055384 | 12.515586 | 8.285485 | 50 |
| FI_male_low | 11.725654 | 2.1547856 | 753 | 7.495553 | 15.955755 | 11.725654 | 50 |
| FI_male_medium | 12.495303 | 0.9395312 | 753 | 10.650892 | 14.339715 | 12.495303 | 263 |

| **Feed intake Gender** | **-Feed intake Gender** | **Difference** | **Std Error** | **t Ratio** | **Prob>\|t\|** | **Lower 95%** | **Upper 95%** |
| --- | --- | --- | --- | --- | --- | --- | --- |
| FI_female_high | FI_female_low | 0.70154 | 3.047327 | 0.23 | 0.8180 | -5.2807 | 6.6838 |
| FI_female_high | FI_female_medium | -0.59471 | 2.352145 | -0.25 | 0.8005 | -5.2123 | 4.0228 |
| FI_female_high | FI_male_high | 5.59633 | 3.047327 | 1.84 | 0.0667 | -0.3859 | 11.5786 |
| FI_female_high | FI_male_low | 2.15616 | 3.047327 | 0.71 | 0.4794 | -3.8261 | 8.1384 |
| FI_female_high | FI_male_medium | 1.38651 | 2.350706 | 0.59 | 0.5555 | -3.2282 | 6.0012 |
| FI_female_low | FI_female_medium | -1.29625 | 2.352145 | -0.55 | 0.5817 | -5.9138 | 3.3213 |
| FI_female_low | FI_male_high | 4.89479 | 3.047327 | 1.61 | 0.1086 | -1.0875 | 10.8771 |
| FI_female_low | FI_male_low | 1.45462 | 3.047327 | 0.48 | 0.6333 | -4.5276 | 7.4369 |
| FI_female_low | FI_male_medium | 0.68497 | 2.350706 | 0.29 | 0.7708 | -3.9297 | 5.2997 |
| FI_female_medium | FI_male_high | 6.19104 | 2.352145 | 2.63 | 0.0087* | 1.5735 | 10.8086 |
| FI_female_medium | FI_male_low | 2.75088 | 2.352145 | 1.17 | 0.2426 | -1.8667 | 7.3684 |
| FI_female_medium | FI_male_medium | 1.98123 | 1.331241 | 1.49 | 0.1371 | -0.6322 | 4.5946 |
| FI_male_high | FI_male_low | -3.44017 | 3.047327 | -1.13 | 0.2593 | -9.4224 | 2.5421 |
| FI_male_high | FI_male_medium | -4.20982 | 2.350706 | -1.79 | 0.0737 | -8.8245 | 0.4049 |
| FI_male_low | FI_male_medium | -0.76965 | 2.350706 | -0.33 | 0.7434 | -5.3844 | 3.8451 |

***Escherichia coli***

| **Feed intake Gender** | **Estimate** | **Std Error** | **DF** | **Lower 95%** | **Upper 95%** | **Arithmetic Mean Estimate** | **N** |
| --- | --- | --- | --- | --- | --- | --- | --- |
| FI_female_high | 9.746420 | 1.3570771 | 753 | 7.0823160 | 12.410525 | 9.746420 | 50 |
| FI_female_low | 12.350082 | 1.3570771 | 753 | 9.6859780 | 15.014187 | 12.350082 | 50 |
| FI_female_medium | 10.339862 | 0.5939765 | 753 | 9.1738151 | 11.505909 | 10.339862 | 261 |
| FI_male_high | 8.998504 | 1.3570771 | 753 | 6.3343997 | 11.662608 | 8.998504 | 50 |
| FI_male_low | 10.553040 | 1.3570771 | 753 | 7.8889358 | 13.217144 | 10.553040 | 50 |
| FI_male_medium | 11.121964 | 0.5917137 | 753 | 9.9603589 | 12.283568 | 11.121964 | 263 |

| **Feed intake Gender** | **-Feed intake Gender** | **Difference** | **Std Error** | **t Ratio** | **Prob>\|t\|** | **Lower 95%** | **Upper 95%** |
| --- | --- | --- | --- | --- | --- | --- | --- |
| FI_female_high | FI_female_low | -2.60366 | 1.919197 | -1.36 | 0.1753 | -6.37127 | 1.163951 |
| FI_female_high | FI_female_medium | -0.59344 | 1.481373 | -0.40 | 0.6888 | -3.50155 | 2.314671 |
| FI_female_high | FI_male_high | 0.74792 | 1.919197 | 0.39 | 0.6969 | -3.01970 | 4.515529 |
| FI_female_high | FI_male_low | -0.80662 | 1.919197 | -0.42 | 0.6744 | -4.57423 | 2.960993 |
| FI_female_high | FI_male_medium | -1.37554 | 1.480467 | -0.93 | 0.3531 | -4.28188 | 1.530791 |
| FI_female_low | FI_female_medium | 2.01022 | 1.481373 | 1.36 | 0.1752 | -0.89789 | 4.918333 |
| FI_female_low | FI_male_high | 3.35158 | 1.919197 | 1.75 | 0.0812 | -0.41603 | 7.119191 |
| FI_female_low | FI_male_low | 1.79704 | 1.919197 | 0.94 | 0.3494 | -1.97057 | 5.564655 |
| FI_female_low | FI_male_medium | 1.22812 | 1.480467 | 0.83 | 0.4071 | -1.67822 | 4.134453 |
| FI_female_medium | FI_male_high | 1.34136 | 1.481373 | 0.91 | 0.3655 | -1.56675 | 4.249470 |
| FI_female_medium | FI_male_low | -0.21318 | 1.481373 | -0.14 | 0.8856 | -3.12129 | 2.694934 |
| FI_female_medium | FI_male_medium | -0.78210 | 0.838411 | -0.93 | 0.3512 | -2.42800 | 0.863799 |
| FI_male_high | FI_male_low | -1.55454 | 1.919197 | -0.81 | 0.4182 | -5.32215 | 2.213076 |
| FI_male_high | FI_male_medium | -2.12346 | 1.480467 | -1.43 | 0.1519 | -5.02979 | 0.782874 |
| FI_male_low | FI_male_medium | -0.56892 | 1.480467 | -0.38 | 0.7009 | -3.47526 | 2.337411 |

***Streptococcus alactolyticus***

| **Feed intake Gender** | **Estimate** | **Std Error** | **DF** | **Lower 95%** | **Upper 95%** | **Arithmetic Mean Estimate** | **N** |
| --- | --- | --- | --- | --- | --- | --- | --- |
| FI_female_high | 4.506045 | 2.0684554 | 753 | 0.445420 | 8.566670 | 4.506045 | 50 |
| FI_female_low | 8.392691 | 2.0684554 | 753 | 4.332066 | 12.453315 | 8.392691 | 50 |
| FI_female_medium | 6.748834 | 0.9053383 | 753 | 4.971546 | 8.526121 | 6.748834 | 261 |
| FI_male_high | 2.742499 | 2.0684554 | 753 | -1.318126 | 6.803124 | 2.742499 | 50 |
| FI_male_low | 12.840554 | 2.0684554 | 753 | 8.779929 | 16.901179 | 12.840554 | 50 |
| FI_male_medium | 9.228499 | 0.9018894 | 753 | 7.457983 | 10.999016 | 9.228499 | 263 |

| **Feed intake Gender** | **-Feed intake Gender** | **Difference** | **Std Error** | **t Ratio** | **Prob>\|t\|** | **Lower 95%** | **Upper 95%** |
| --- | --- | --- | --- | --- | --- | --- | --- |
| FI_female_high | FI_female_low | -3.8866 | 2.925238 | -1.33 | 0.1844 | -9.6292 | 1.8559 |
| FI_female_high | FI_female_medium | -2.2428 | 2.257907 | -0.99 | 0.3209 | -6.6753 | 2.1898 |
| FI_female_high | FI_male_high | 1.7635 | 2.925238 | 0.60 | 0.5468 | -3.9790 | 7.5061 |
| FI_female_high | FI_male_low | -8.3345 | 2.925238 | -2.85 | 0.0045* | -14.0771 | -2.5919 |
| FI_female_high | FI_male_medium | -4.7225 | 2.256527 | -2.09 | 0.0367* | -9.1523 | -0.2926 |
| FI_female_low | FI_female_medium | 1.6439 | 2.257907 | 0.73 | 0.4668 | -2.7887 | 6.0764 |
| FI_female_low | FI_male_high | 5.6502 | 2.925238 | 1.93 | 0.0538 | -0.0924 | 11.3928 |
| FI_female_low | FI_male_low | -4.4479 | 2.925238 | -1.52 | 0.1288 | -10.1905 | 1.2947 |
| FI_female_low | FI_male_medium | -0.8358 | 2.256527 | -0.37 | 0.7112 | -5.2656 | 3.5940 |
| FI_female_medium | FI_male_high | 4.0063 | 2.257907 | 1.77 | 0.0764 | -0.4262 | 8.4389 |
| FI_female_medium | FI_male_low | -6.0917 | 2.257907 | -2.70 | 0.0071* | -10.5243 | -1.6592 |
| FI_female_medium | FI_male_medium | -2.4797 | 1.277905 | -1.94 | 0.0527 | -4.9883 | 0.0290 |
| FI_male_high | FI_male_low | -10.0981 | 2.925238 | -3.45 | 0.0006* | -15.8406 | -4.3555 |
| FI_male_high | FI_male_medium | -6.4860 | 2.256527 | -2.87 | 0.0042* | -10.9158 | -2.0562 |
| FI_male_low | FI_male_medium | 3.6121 | 2.256527 | 1.60 | 0.1099 | -0.8178 | 8.0419 |

***Enterococcus faecium***

| **Feed intake Gender** | **Estimate** | **Std Error** | **DF** | **Lower 95%** | **Upper 95%** | **Arithmetic Mean Estimate** | **N** |
| --- | --- | --- | --- | --- | --- | --- | --- |
| FI_female_high | 1.0165278 | 0.76222781 | 753 | -0.479816 | 2.5128720 | 1.0165278 | 50 |
| FI_female_low | 3.3210065 | 0.76222781 | 753 | 1.824662 | 4.8173507 | 3.3210065 | 50 |
| FI_female_medium | 0.9544495 | 0.33361805 | 753 | 0.299517 | 1.6093815 | 0.9544495 | 261 |
| FI_male_high | 1.5457418 | 0.76222781 | 753 | 0.049398 | 3.0420860 | 1.5457418 | 50 |
| FI_male_low | 2.4312563 | 0.76222781 | 753 | 0.934912 | 3.9276005 | 2.4312563 | 50 |
| FI_male_medium | 1.6806718 | 0.33234712 | 753 | 1.028235 | 2.3331089 | 1.6806718 | 263 |

| **Feed intake Gender** | **-Feed intake Gender** | **Difference** | **Std Error** | **t Ratio** | **Prob>\|t\|** | **Lower 95%** | **Upper 95%** |
| --- | --- | --- | --- | --- | --- | --- | --- |
| FI_female_high | FI_female_low | -2.30448 | 1.077953 | -2.14 | 0.0329* | -4.42063 | -0.18833 |
| FI_female_high | FI_female_medium | 0.06208 | 0.832041 | 0.07 | 0.9405 | -1.57132 | 1.69547 |
| FI_female_high | FI_male_high | -0.52921 | 1.077953 | -0.49 | 0.6236 | -2.64536 | 1.58694 |
| FI_female_high | FI_male_low | -1.41473 | 1.077953 | -1.31 | 0.1898 | -3.53088 | 0.70142 |
| FI_female_high | FI_male_medium | -0.66414 | 0.831532 | -0.80 | 0.4247 | -2.29654 | 0.96825 |
| FI_female_low | FI_female_medium | 2.36656 | 0.832041 | 2.84 | 0.0046* | 0.73316 | 3.99995 |
| FI_female_low | FI_male_high | 1.77526 | 1.077953 | 1.65 | 0.1000 | -0.34089 | 3.89141 |
| FI_female_low | FI_male_low | 0.88975 | 1.077953 | 0.83 | 0.4094 | -1.22640 | 3.00590 |
| FI_female_low | FI_male_medium | 1.64033 | 0.831532 | 1.97 | 0.0489* | 0.00794 | 3.27273 |
| FI_female_medium | FI_male_high | -0.59129 | 0.832041 | -0.71 | 0.4775 | -2.22469 | 1.04210 |
| FI_female_medium | FI_male_low | -1.47681 | 0.832041 | -1.77 | 0.0763 | -3.11020 | 0.15659 |
| FI_female_medium | FI_male_medium | -0.72622 | 0.470909 | -1.54 | 0.1235 | -1.65067 | 0.19823 |
| FI_male_high | FI_male_low | -0.88551 | 1.077953 | -0.82 | 0.4116 | -3.00166 | 1.23064 |
| FI_male_high | FI_male_medium | -0.13493 | 0.831532 | -0.16 | 0.8711 | -1.76733 | 1.49747 |
| FI_male_low | FI_male_medium | 0.75058 | 0.831532 | 0.90 | 0.3670 | -0.88181 | 2.38298 |

**D- Body weight gain**

**Unclassified Clostridiaceae1**

| **BWG Gender** | **Estimate** | **Std Error** | **DF** | **Lower 95%** | **Upper 95%** | **Arithmetic Mean Estimate** | **N** |
| --- | --- | --- | --- | --- | --- | --- | --- |
| BWG_female_high | 36.052649 | 2.5686635 | 753 | 31.010056 | 41.095242 | 36.052649 | 50 |
| BWG_female_low | 26.244438 | 2.5686635 | 753 | 21.201845 | 31.287031 | 26.244438 | 50 |
| BWG_female_medium | 27.725387 | 1.1242735 | 753 | 25.518304 | 29.932470 | 27.725387 | 261 |
| BWG_male_high | 43.160369 | 2.5686635 | 753 | 38.117776 | 48.202962 | 43.160369 | 50 |
| BWG_male_low | 22.556769 | 2.5686635 | 753 | 17.514176 | 27.599362 | 22.556769 | 50 |
| BWG_male_medium | 29.599747 | 1.1199905 | 753 | 27.401072 | 31.798422 | 29.599747 | 263 |

| **BWG Gender** | **-BWG Gender** | **Difference** | **Std Error** | **t Ratio** | **Prob>\|t\|** | **Lower 95%** | **Upper 95%** |
| --- | --- | --- | --- | --- | --- | --- | --- |
| BWG_female_high | BWG_female_low | 9.8082 | 3.632639 | 2.70 | 0.0071* | 2.6769 | 16.9395 |
| BWG_female_high | BWG_female_medium | 8.3273 | 2.803930 | 2.97 | 0.0031* | 2.8228 | 13.8317 |
| BWG_female_high | BWG_male_high | -7.1077 | 3.632639 | -1.96 | 0.0508 | -14.2390 | 0.0236 |
| BWG_female_high | BWG_male_low | 13.4959 | 3.632639 | 3.72 | 0.0002* | 6.3646 | 20.6272 |
| BWG_female_high | BWG_male_medium | 6.4529 | 2.802215 | 2.30 | 0.0216* | 0.9518 | 11.9540 |
| BWG_female_low | BWG_female_medium | -1.4809 | 2.803930 | -0.53 | 0.5975 | -6.9854 | 4.0235 |
| BWG_female_low | BWG_male_high | -16.9159 | 3.632639 | -4.66 | <.0001* | -24.0472 | -9.7846 |
| BWG_female_low | BWG_male_low | 3.6877 | 3.632639 | 1.02 | 0.3104 | -3.4436 | 10.8190 |
| BWG_female_low | BWG_male_medium | -3.3553 | 2.802215 | -1.20 | 0.2315 | -8.8564 | 2.1458 |
| BWG_female_medium | BWG_male_high | -15.4350 | 2.803930 | -5.50 | <.0001* | -20.9394 | -9.9305 |
| BWG_female_medium | BWG_male_low | 5.1686 | 2.803930 | 1.84 | 0.0657 | -0.3358 | 10.6731 |
| BWG_female_medium | BWG_male_medium | -1.8744 | 1.586937 | -1.18 | 0.2379 | -4.9897 | 1.2410 |
| BWG_male_high | BWG_male_low | 20.6036 | 3.632639 | 5.67 | <.0001* | 13.4723 | 27.7349 |
| BWG_male_high | BWG_male_medium | 13.5606 | 2.802215 | 4.84 | <.0001* | 8.0595 | 19.0617 |
| BWG_male_low | BWG_male_medium | -7.0430 | 2.802215 | -2.51 | 0.0122* | -12.5441 | -1.5419 |

**Unclassified *Lactobacillus***

| **BWG Gender** | **Estimate** | **Std Error** | **DF** | **Lower 95%** | **Upper 95%** | **Arithmetic Mean Estimate** | **N** |
| --- | --- | --- | --- | --- | --- | --- | --- |
| BWG_female_high | 16.594142 | 2.8372794 | 753 | 11.024224 | 22.164060 | 16.594142 | 50 |
| BWG_female_low | 22.386865 | 2.8372794 | 753 | 16.816947 | 27.956784 | 22.386865 | 50 |
| BWG_female_medium | 26.449348 | 1.2418434 | 753 | 24.011461 | 28.887235 | 26.449348 | 261 |
| BWG_male_high | 16.367436 | 2.8372794 | 753 | 10.797518 | 21.937355 | 16.367436 | 50 |
| BWG_male_low | 21.449781 | 2.8372794 | 753 | 15.879863 | 27.019700 | 21.449781 | 50 |
| BWG_male_medium | 21.269347 | 1.2371126 | 753 | 18.840747 | 23.697947 | 21.269347 | 263 |

| **BWG Gender** | **-BWG Gender** | **Difference** | **Std Error** | **t Ratio** | **Prob>\|t\|** | **Lower 95%** | **Upper 95%** |
| --- | --- | --- | --- | --- | --- | --- | --- |
| BWG_female_high | BWG_female_low | -5.7927 | 4.012519 | -1.44 | 0.1492 | -13.6698 | 2.0843 |
| BWG_female_high | BWG_female_medium | -9.8552 | 3.097149 | -3.18 | 0.0015* | -15.9353 | -3.7751 |
| BWG_female_high | BWG_male_high | 0.2267 | 4.012519 | 0.06 | 0.9550 | -7.6503 | 8.1038 |
| BWG_female_high | BWG_male_low | -4.8556 | 4.012519 | -1.21 | 0.2266 | -12.7327 | 3.0214 |
| BWG_female_high | BWG_male_medium | -4.6752 | 3.095255 | -1.51 | 0.1314 | -10.7516 | 1.4011 |
| BWG_female_low | BWG_female_medium | -4.0625 | 3.097149 | -1.31 | 0.1900 | -10.1426 | 2.0176 |
| BWG_female_low | BWG_male_high | 6.0194 | 4.012519 | 1.50 | 0.1340 | -1.8576 | 13.8965 |
| BWG_female_low | BWG_male_low | 0.9371 | 4.012519 | 0.23 | 0.8154 | -6.9400 | 8.8141 |
| BWG_female_low | BWG_male_medium | 1.1175 | 3.095255 | 0.36 | 0.7182 | -4.9588 | 7.1939 |
| BWG_female_medium | BWG_male_high | 10.0819 | 3.097149 | 3.26 | 0.0012* | 4.0018 | 16.1620 |
| BWG_female_medium | BWG_male_low | 4.9996 | 3.097149 | 1.61 | 0.1069 | -1.0805 | 11.0796 |
| BWG_female_medium | BWG_male_medium | 5.1800 | 1.752890 | 2.96 | 0.0032* | 1.7389 | 8.6211 |
| BWG_male_high | BWG_male_low | -5.0823 | 4.012519 | -1.27 | 0.2057 | -12.9594 | 2.7947 |
| BWG_male_high | BWG_male_medium | -4.9019 | 3.095255 | -1.58 | 0.1137 | -10.9783 | 1.1744 |
| BWG_male_low | BWG_male_medium | 0.1804 | 3.095255 | 0.06 | 0.9535 | -5.8959 | 6.2568 |

**Unclassified *Clostridium* sensu stricto 1**

| **BWG Gender** | **Estimate** | **Std Error** | **DF** | **Lower 95%** | **Upper 95%** | **Arithmetic Mean Estimate** | **N** |
| --- | --- | --- | --- | --- | --- | --- | --- |
| BWG_female_high | 13.620069 | 2.1511890 | 753 | 9.397028 | 17.843110 | 13.620069 | 50 |
| BWG_female_low | 10.588467 | 2.1511890 | 753 | 6.365426 | 14.811508 | 10.588467 | 50 |
| BWG_female_medium | 15.023187 | 0.9415499 | 753 | 13.174813 | 16.871562 | 15.023187 | 261 |
| BWG_male_high | 9.974806 | 2.1511890 | 753 | 5.751765 | 14.197847 | 9.974806 | 50 |
| BWG_male_low | 9.629042 | 2.1511890 | 753 | 5.406001 | 13.852083 | 9.629042 | 50 |
| BWG_male_medium | 12.572735 | 0.9379630 | 753 | 10.731402 | 14.414069 | 12.572735 | 263 |

| **BWG Gender** | **-BWG Gender** | **Difference** | **Std Error** | **t Ratio** | **Prob>\|t\|** | **Lower 95%** | **Upper 95%** |
| --- | --- | --- | --- | --- | --- | --- | --- |
| BWG_female_high | BWG_female_low | 3.03160 | 3.042241 | 1.00 | 0.3193 | -2.9407 | 9.0039 |
| BWG_female_high | BWG_female_medium | -1.40312 | 2.348219 | -0.60 | 0.5503 | -6.0130 | 3.2067 |
| BWG_female_high | BWG_male_high | 3.64526 | 3.042241 | 1.20 | 0.2312 | -2.3270 | 9.6175 |
| BWG_female_high | BWG_male_low | 3.99103 | 3.042241 | 1.31 | 0.1900 | -1.9813 | 9.9633 |
| BWG_female_high | BWG_male_medium | 1.04733 | 2.346783 | 0.45 | 0.6555 | -3.5597 | 5.6543 |
| BWG_female_low | BWG_female_medium | -4.43472 | 2.348219 | -1.89 | 0.0593 | -9.0446 | 0.1751 |
| BWG_female_low | BWG_male_high | 0.61366 | 3.042241 | 0.20 | 0.8402 | -5.3586 | 6.5859 |
| BWG_female_low | BWG_male_low | 0.95942 | 3.042241 | 0.32 | 0.7526 | -5.0129 | 6.9317 |
| BWG_female_low | BWG_male_medium | -1.98427 | 2.346783 | -0.85 | 0.3981 | -6.5913 | 2.6227 |
| BWG_female_medium | BWG_male_high | 5.04838 | 2.348219 | 2.15 | 0.0319* | 0.4385 | 9.6582 |
| BWG_female_medium | BWG_male_low | 5.39415 | 2.348219 | 2.30 | 0.0219* | 0.7843 | 10.0040 |
| BWG_female_medium | BWG_male_medium | 2.45045 | 1.329019 | 1.84 | 0.0656 | -0.1586 | 5.0595 |
| BWG_male_high | BWG_male_low | 0.34576 | 3.042241 | 0.11 | 0.9095 | -5.6265 | 6.3180 |
| BWG_male_high | BWG_male_medium | -2.59793 | 2.346783 | -1.11 | 0.2686 | -7.2049 | 2.0091 |
| BWG_male_low | BWG_male_medium | -2.94369 | 2.346783 | -1.25 | 0.2101 | -7.5507 | 1.6633 |

***Escherichia coli***

| **BWG Gender** | **Estimate** | **Std Error** | **DF** | **Lower 95%** | **Upper 95%** | **Arithmetic Mean Estimate** | **N** |
| --- | --- | --- | --- | --- | --- | --- | --- |
| BWG_female_high | 10.230986 | 1.3592326 | 753 | 7.5626500 | 12.899322 | 10.230986 | 50 |
| BWG_female_low | 11.165203 | 1.3592326 | 753 | 8.4968669 | 13.833539 | 11.165203 | 50 |
| BWG_female_medium | 10.474022 | 0.5949199 | 753 | 9.3061229 | 11.641921 | 10.474022 | 261 |
| BWG_male_high | 10.062772 | 1.3592326 | 753 | 7.3944360 | 12.731108 | 10.062772 | 50 |
| BWG_male_low | 9.416888 | 1.3592326 | 753 | 6.7485526 | 12.085224 | 9.416888 | 50 |
| BWG_male_medium | 11.135630 | 0.5926536 | 753 | 9.9721801 | 12.299080 | 11.135630 | 263 |

| **BWG Gender** | **-BWG Gender** | **Difference** | **Std Error** | **t Ratio** | **Prob>\|t\|** | **Lower 95%** | **Upper 95%** |
| --- | --- | --- | --- | --- | --- | --- | --- |
| BWG_female_high | BWG_female_low | -0.93422 | 1.922245 | -0.49 | 0.6271 | -4.70781 | 2.839380 |
| BWG_female_high | BWG_female_medium | -0.24304 | 1.483726 | -0.16 | 0.8699 | -3.15577 | 2.669695 |
| BWG_female_high | BWG_male_high | 0.16821 | 1.922245 | 0.09 | 0.9303 | -3.60538 | 3.941811 |
| BWG_female_high | BWG_male_low | 0.81410 | 1.922245 | 0.42 | 0.6720 | -2.95950 | 4.587694 |
| BWG_female_high | BWG_male_medium | -0.90464 | 1.482819 | -0.61 | 0.5420 | -3.81559 | 2.006306 |
| BWG_female_low | BWG_female_medium | 0.69118 | 1.483726 | 0.47 | 0.6415 | -2.22155 | 3.603912 |
| BWG_female_low | BWG_male_high | 1.10243 | 1.922245 | 0.57 | 0.5665 | -2.67117 | 4.876028 |
| BWG_female_low | BWG_male_low | 1.74831 | 1.922245 | 0.91 | 0.3634 | -2.02528 | 5.521911 |
| BWG_female_low | BWG_male_medium | 0.02957 | 1.482819 | 0.02 | 0.9841 | -2.88138 | 2.940523 |
| BWG_female_medium | BWG_male_high | 0.41125 | 1.483726 | 0.28 | 0.7817 | -2.50148 | 3.323981 |
| BWG_female_medium | BWG_male_low | 1.05713 | 1.483726 | 0.71 | 0.4764 | -1.85560 | 3.969865 |
| BWG_female_medium | BWG_male_medium | -0.66161 | 0.839743 | -0.79 | 0.4310 | -2.31012 | 0.986907 |
| BWG_male_high | BWG_male_low | 0.64588 | 1.922245 | 0.34 | 0.7370 | -3.12771 | 4.419480 |
| BWG_male_high | BWG_male_medium | -1.07286 | 1.482819 | -0.72 | 0.4696 | -3.98381 | 1.838092 |
| BWG_male_low | BWG_male_medium | -1.71874 | 1.482819 | -1.16 | 0.2468 | -4.62969 | 1.192209 |

***Streptococcus alactolyticus***

| **BWG Gender** | **Estimate** | **Std Error** | **DF** | **Lower 95%** | **Upper 95%** | **Arithmetic Mean Estimate** | **N** |
| --- | --- | --- | --- | --- | --- | --- | --- |
| BWG_female_high | 6.312642 | 2.0611330 | 753 | 2.266392 | 10.358892 | 6.312642 | 50 |
| BWG_female_low | 8.417384 | 2.0611330 | 753 | 4.371134 | 12.463634 | 8.417384 | 50 |
| BWG_female_medium | 6.398012 | 0.9021334 | 753 | 4.627016 | 8.169007 | 6.398012 | 261 |
| BWG_male_high | 4.069952 | 2.0611330 | 753 | 0.023701 | 8.116202 | 4.069952 | 50 |
| BWG_male_low | 16.364045 | 2.0611330 | 753 | 12.317795 | 20.410295 | 16.364045 | 50 |
| BWG_male_medium | 8.306267 | 0.8986967 | 753 | 6.542018 | 10.070516 | 8.306267 | 263 |

| **BWG Gender** | **-BWG Gender** | **Difference** | **Std Error** | **t Ratio** | **Prob>\|t\|** | **Lower 95%** | **Upper 95%** |
| --- | --- | --- | --- | --- | --- | --- | --- |
| BWG_female_high | BWG_female_low | -2.1047 | 2.914882 | -0.72 | 0.4705 | -7.8270 | 3.6175 |
| BWG_female_high | BWG_female_medium | -0.0854 | 2.249914 | -0.04 | 0.9697 | -4.5022 | 4.3315 |
| BWG_female_high | BWG_male_high | 2.2427 | 2.914882 | 0.77 | 0.4419 | -3.4796 | 7.9650 |
| BWG_female_high | BWG_male_low | -10.0514 | 2.914882 | -3.45 | 0.0006* | -15.7737 | -4.3291 |
| BWG_female_high | BWG_male_medium | -1.9936 | 2.248538 | -0.89 | 0.3756 | -6.4078 | 2.4205 |
| BWG_female_low | BWG_female_medium | 2.0194 | 2.249914 | 0.90 | 0.3697 | -2.3975 | 6.4362 |
| BWG_female_low | BWG_male_high | 4.3474 | 2.914882 | 1.49 | 0.1363 | -1.3748 | 10.0697 |
| BWG_female_low | BWG_male_low | -7.9467 | 2.914882 | -2.73 | 0.0066* | -13.6689 | -2.2244 |
| BWG_female_low | BWG_male_medium | 0.1111 | 2.248538 | 0.05 | 0.9606 | -4.3030 | 4.5253 |
| BWG_female_medium | BWG_male_high | 2.3281 | 2.249914 | 1.03 | 0.3011 | -2.0888 | 6.7449 |
| BWG_female_medium | BWG_male_low | -9.9660 | 2.249914 | -4.43 | <.0001* | -14.3829 | -5.5492 |
| BWG_female_medium | BWG_male_medium | -1.9083 | 1.273381 | -1.50 | 0.1344 | -4.4081 | 0.5915 |
| BWG_male_high | BWG_male_low | -12.2941 | 2.914882 | -4.22 | <.0001* | -18.0164 | -6.5718 |
| BWG_male_high | BWG_male_medium | -4.2363 | 2.248538 | -1.88 | 0.0599 | -8.6505 | 0.1778 |
| BWG_male_low | BWG_male_medium | 8.0578 | 2.248538 | 3.58 | 0.0004* | 3.6436 | 12.4719 |

***Enterococcus faecium***

| **BWG Gender** | **Estimate** | **Std Error** | **DF** | **Lower 95%** | **Upper 95%** | **Arithmetic Mean Estimate** | **N** |
| --- | --- | --- | --- | --- | --- | --- | --- |
| BWG_female_high | 1.0074827 | 0.76302966 | 753 | -0.490436 | 2.5054010 | 1.0074827 | 50 |
| BWG_female_low | 2.7617785 | 0.76302966 | 753 | 1.263860 | 4.2596968 | 2.7617785 | 50 |
| BWG_female_medium | 1.0633141 | 0.33396901 | 753 | 0.407693 | 1.7189351 | 1.0633141 | 261 |
| BWG_male_high | 2.5964289 | 0.76302966 | 753 | 1.098511 | 4.0943472 | 2.5964289 | 50 |
| BWG_male_low | 2.5857747 | 0.76302966 | 753 | 1.087856 | 4.0836930 | 2.5857747 | 50 |
| BWG_male_medium | 1.4515453 | 0.33269674 | 753 | 0.798422 | 2.1046687 | 1.4515453 | 263 |

| **BWG Gender** | **-BWG Gender** | **Difference** | **Std Error** | **t Ratio** | **Prob>\|t\|** | **Lower 95%** | **Upper 95%** |
| --- | --- | --- | --- | --- | --- | --- | --- |
| BWG_female_high | BWG_female_low | -1.75430 | 1.079087 | -1.63 | 0.1044 | -3.87267 | 0.364081 |
| BWG_female_high | BWG_female_medium | -0.05583 | 0.832916 | -0.07 | 0.9466 | -1.69095 | 1.579283 |
| BWG_female_high | BWG_male_high | -1.58895 | 1.079087 | -1.47 | 0.1413 | -3.70732 | 0.529430 |
| BWG_female_high | BWG_male_low | -1.57829 | 1.079087 | -1.46 | 0.1440 | -3.69667 | 0.540084 |
| BWG_female_high | BWG_male_medium | -0.44406 | 0.832407 | -0.53 | 0.5939 | -2.07818 | 1.190052 |
| BWG_female_low | BWG_female_medium | 1.69846 | 0.832916 | 2.04 | 0.0418* | 0.06335 | 3.333579 |
| BWG_female_low | BWG_male_high | 0.16535 | 1.079087 | 0.15 | 0.8783 | -1.95303 | 2.283726 |
| BWG_female_low | BWG_male_low | 0.17600 | 1.079087 | 0.16 | 0.8705 | -1.94237 | 2.294380 |
| BWG_female_low | BWG_male_medium | 1.31023 | 0.832407 | 1.57 | 0.1159 | -0.32388 | 2.944348 |
| BWG_female_medium | BWG_male_high | -1.53311 | 0.832916 | -1.84 | 0.0661 | -3.16823 | 0.101999 |
| BWG_female_medium | BWG_male_low | -1.52246 | 0.832916 | -1.83 | 0.0680 | -3.15757 | 0.112654 |
| BWG_female_medium | BWG_male_medium | -0.38823 | 0.471405 | -0.82 | 0.4104 | -1.31366 | 0.537193 |
| BWG_male_high | BWG_male_low | 0.01065 | 1.079087 | 0.01 | 0.9921 | -2.10772 | 2.129031 |
| BWG_male_high | BWG_male_medium | 1.14488 | 0.832407 | 1.38 | 0.1694 | -0.48923 | 2.778998 |
| BWG_male_low | BWG_male_medium | 1.13423 | 0.832407 | 1.36 | 0.1734 | -0.49988 | 2.768344 |

**E- Feed Conversion**

**Unclassified Clostridiaceae1**

| **Feed conversion Gender** | **Estimate** | **Std Error** | **DF** | **Lower 95%** | **Upper 95%** | **Arithmetic Mean Estimate** | **N** |
| --- | --- | --- | --- | --- | --- | --- | --- |
| FC_female_high | 29.785504 | 2.6370708 | 753 | 24.608619 | 34.962388 | 29.785504 | 50 |
| FC_female_low | 24.718322 | 2.6370708 | 753 | 19.541437 | 29.895207 | 24.718322 | 50 |
| FC_female_medium | 29.218349 | 1.1542145 | 753 | 26.952488 | 31.484210 | 29.218349 | 261 |
| FC_male_high | 31.859148 | 2.6370708 | 753 | 26.682263 | 37.036033 | 31.859148 | 50 |
| FC_male_low | 28.456622 | 2.6370708 | 753 | 23.279737 | 33.633507 | 28.456622 | 50 |
| FC_male_medium | 30.626623 | 1.1498175 | 753 | 28.369394 | 32.883852 | 30.626623 | 263 |

| **Feed conversion Gender** | **-Feed conversion Gender** | **Difference** | **Std Error** | **t Ratio** | **Prob>\|t\|** | **Lower 95%** | **Upper 95%** |
| --- | --- | --- | --- | --- | --- | --- | --- |
| FC_female_high | FC_female_low | 5.06718 | 3.729381 | 1.36 | 0.1746 | -2.2540 | 12.3884 |
| FC_female_high | FC_female_medium | 0.56715 | 2.878603 | 0.20 | 0.8439 | -5.0839 | 6.2182 |
| FC_female_high | FC_male_high | -2.07364 | 3.729381 | -0.56 | 0.5784 | -9.3949 | 5.2476 |
| FC_female_high | FC_male_low | 1.32888 | 3.729381 | 0.36 | 0.7217 | -5.9923 | 8.6501 |
| FC_female_high | FC_male_medium | -0.84112 | 2.876842 | -0.29 | 0.7701 | -6.4887 | 4.8065 |
| FC_female_low | FC_female_medium | -4.50003 | 2.878603 | -1.56 | 0.1184 | -10.1511 | 1.1510 |
| FC_female_low | FC_male_high | -7.14083 | 3.729381 | -1.91 | 0.0559 | -14.4620 | 0.1804 |
| FC_female_low | FC_male_low | -3.73830 | 3.729381 | -1.00 | 0.3165 | -11.0595 | 3.5829 |
| FC_female_low | FC_male_medium | -5.90830 | 2.876842 | -2.05 | 0.0403* | -11.5559 | -0.2607 |
| FC_female_medium | FC_male_high | -2.64080 | 2.878603 | -0.92 | 0.3592 | -8.2918 | 3.0102 |
| FC_female_medium | FC_male_low | 0.76173 | 2.878603 | 0.26 | 0.7914 | -4.8893 | 6.4128 |
| FC_female_medium | FC_male_medium | -1.40827 | 1.629200 | -0.86 | 0.3876 | -4.6066 | 1.7900 |
| FC_male_high | FC_male_low | 3.40253 | 3.729381 | 0.91 | 0.3619 | -3.9187 | 10.7237 |
| FC_male_high | FC_male_medium | 1.23252 | 2.876842 | 0.43 | 0.6685 | -4.4151 | 6.8801 |
| FC_male_low | FC_male_medium | -2.17000 | 2.876842 | -0.75 | 0.4509 | -7.8176 | 3.4776 |

**Unclassified *Lactobacillus***

| **Feed conversion Gender** | **Estimate** | **Std Error** | **DF** | **Lower 95%** | **Upper 95%** | **Arithmetic Mean Estimate** | **N** |
| --- | --- | --- | --- | --- | --- | --- | --- |
| FC_female_high | 21.725422 | 2.8453779 | 753 | 16.139605 | 27.311238 | 21.725422 | 50 |
| FC_female_low | 26.430286 | 2.8453779 | 753 | 20.844469 | 32.016102 | 26.430286 | 50 |
| FC_female_medium | 24.691743 | 1.2453881 | 753 | 22.246897 | 27.136588 | 24.691743 | 261 |
| FC_male_high | 13.335501 | 2.8453779 | 753 | 7.749685 | 18.921318 | 13.335501 | 50 |
| FC_male_low | 21.761135 | 2.8453779 | 753 | 16.175319 | 27.346952 | 21.761135 | 50 |
| FC_male_medium | 21.786568 | 1.2406437 | 753 | 19.351036 | 24.222099 | 21.786568 | 263 |

| **Feed conversion Gender** | **-Feed conversion Gender** | **Difference** | **Std Error** | **t Ratio** | **Prob>\|t\|** | **Lower 95%** | **Upper 95%** |
| --- | --- | --- | --- | --- | --- | --- | --- |
| FC_female_high | FC_female_low | -4.7049 | 4.023972 | -1.17 | 0.2427 | -12.6044 | 3.1947 |
| FC_female_high | FC_female_medium | -2.9663 | 3.105989 | -0.96 | 0.3399 | -9.0637 | 3.1311 |
| FC_female_high | FC_male_high | 8.3899 | 4.023972 | 2.08 | 0.0374* | 0.4904 | 16.2895 |
| FC_female_high | FC_male_low | -0.0357 | 4.023972 | -0.01 | 0.9929 | -7.9353 | 7.8638 |
| FC_female_high | FC_male_medium | -0.0611 | 3.104090 | -0.02 | 0.9843 | -6.1548 | 6.0326 |
| FC_female_low | FC_female_medium | 1.7385 | 3.105989 | 0.56 | 0.5758 | -4.3589 | 7.8360 |
| FC_female_low | FC_male_high | 13.0948 | 4.023972 | 3.25 | 0.0012* | 5.1952 | 20.9943 |
| FC_female_low | FC_male_low | 4.6692 | 4.023972 | 1.16 | 0.2463 | -3.2304 | 12.5687 |
| FC_female_low | FC_male_medium | 4.6437 | 3.104090 | 1.50 | 0.1351 | -1.4500 | 10.7374 |
| FC_female_medium | FC_male_high | 11.3562 | 3.105989 | 3.66 | 0.0003* | 5.2588 | 17.4537 |
| FC_female_medium | FC_male_low | 2.9306 | 3.105989 | 0.94 | 0.3457 | -3.1668 | 9.0280 |
| FC_female_medium | FC_male_medium | 2.9052 | 1.757893 | 1.65 | 0.0988 | -0.5458 | 6.3561 |
| FC_male_high | FC_male_low | -8.4256 | 4.023972 | -2.09 | 0.0366* | -16.3252 | -0.5261 |
| FC_male_high | FC_male_medium | -8.4511 | 3.104090 | -2.72 | 0.0066* | -14.5448 | -2.3574 |
| FC_male_low | FC_male_medium | -0.0254 | 3.104090 | -0.01 | 0.9935 | -6.1191 | 6.0683 |

**Unclassified *Clostridium* sensu stricto 1**

| **Feed conversion Gender** | **Estimate** | **Std Error** | **DF** | **Lower 95%** | **Upper 95%** | **Arithmetic Mean Estimate** | **N** |
| --- | --- | --- | --- | --- | --- | --- | --- |
| FC_female_high | 10.549844 | 2.1527127 | 753 | 6.323812 | 14.775876 | 10.549844 | 50 |
| FC_female_low | 14.964459 | 2.1527127 | 753 | 10.738427 | 19.190491 | 14.964459 | 50 |
| FC_female_medium | 14.773041 | 0.9422168 | 753 | 12.923357 | 16.622725 | 14.773041 | 261 |
| FC_male_high | 9.365294 | 2.1527127 | 753 | 5.139262 | 13.591326 | 9.365294 | 50 |
| FC_male_low | 12.917624 | 2.1527127 | 753 | 8.691592 | 17.143656 | 12.917624 | 50 |
| FC_male_medium | 12.063406 | 0.9386273 | 753 | 10.220769 | 13.906044 | 12.063406 | 263 |

| **Feed conversion Gender** | **-Feed conversion Gender** | **Difference** | **Std Error** | **t Ratio** | **Prob>\|t\|** | **Lower 95%** | **Upper 95%** |
| --- | --- | --- | --- | --- | --- | --- | --- |
| FC_female_high | FC_female_low | -4.41461 | 3.044396 | -1.45 | 0.1475 | -10.3911 | 1.5619 |
| FC_female_high | FC_female_medium | -4.22320 | 2.349882 | -1.80 | 0.0727 | -8.8363 | 0.3899 |
| FC_female_high | FC_male_high | 1.18455 | 3.044396 | 0.39 | 0.6973 | -4.7920 | 7.1611 |
| FC_female_high | FC_male_low | -2.36778 | 3.044396 | -0.78 | 0.4370 | -8.3443 | 3.6087 |
| FC_female_high | FC_male_medium | -1.51356 | 2.348445 | -0.64 | 0.5195 | -6.1238 | 3.0967 |
| FC_female_low | FC_female_medium | 0.19142 | 2.349882 | 0.08 | 0.9351 | -4.4217 | 4.8045 |
| FC_female_low | FC_male_high | 5.59916 | 3.044396 | 1.84 | 0.0663 | -0.3773 | 11.5757 |
| FC_female_low | FC_male_low | 2.04683 | 3.044396 | 0.67 | 0.5016 | -3.9297 | 8.0233 |
| FC_female_low | FC_male_medium | 2.90105 | 2.348445 | 1.24 | 0.2171 | -1.7092 | 7.5113 |
| FC_female_medium | FC_male_high | 5.40775 | 2.349882 | 2.30 | 0.0216* | 0.7946 | 10.0208 |
| FC_female_medium | FC_male_low | 1.85542 | 2.349882 | 0.79 | 0.4300 | -2.7577 | 6.4685 |
| FC_female_medium | FC_male_medium | 2.70963 | 1.329960 | 2.04 | 0.0420* | 0.0988 | 5.3205 |
| FC_male_high | FC_male_low | -3.55233 | 3.044396 | -1.17 | 0.2436 | -9.5288 | 2.4242 |
| FC_male_high | FC_male_medium | -2.69811 | 2.348445 | -1.15 | 0.2510 | -7.3084 | 1.9122 |
| FC_male_low | FC_male_medium | 0.85422 | 2.348445 | 0.36 | 0.7162 | -3.7561 | 5.4645 |

***Escherichia coli***

| **Feed conversion Gender** | **Estimate** | **Std Error** | **DF** | **Lower 95%** | **Upper 95%** | **Arithmetic Mean Estimate** | **N** |
| --- | --- | --- | --- | --- | --- | --- | --- |
| FC_female_high | 9.016991 | 1.3506857 | 753 | 6.365434 | 11.668548 | 9.016991 | 50 |
| FC_female_low | 12.771244 | 1.3506857 | 753 | 10.119687 | 15.422802 | 12.771244 | 50 |
| FC_female_medium | 10.398917 | 0.5911791 | 753 | 9.238362 | 11.559472 | 10.398917 | 261 |
| FC_male_high | 9.071459 | 1.3506857 | 753 | 6.419901 | 11.723016 | 9.071459 | 50 |
| FC_male_low | 13.962373 | 1.3506857 | 753 | 11.310816 | 16.613931 | 13.962373 | 50 |
| FC_male_medium | 10.459932 | 0.5889269 | 753 | 9.303798 | 11.616066 | 10.459932 | 263 |

| **Feed conversion Gender** | **-Feed conversion Gender** | **Difference** | **Std Error** | **t Ratio** | **Prob>\|t\|** | **Lower 95%** | **Upper 95%** |
| --- | --- | --- | --- | --- | --- | --- | --- |
| FC_female_high | FC_female_low | -3.75425 | 1.910158 | -1.97 | 0.0497* | -7.50412 | -0.00439 |
| FC_female_high | FC_female_medium | -1.38193 | 1.474396 | -0.94 | 0.3489 | -4.27634 | 1.51249 |
| FC_female_high | FC_male_high | -0.05447 | 1.910158 | -0.03 | 0.9773 | -3.80434 | 3.69540 |
| FC_female_high | FC_male_low | -4.94538 | 1.910158 | -2.59 | 0.0098* | -8.69525 | -1.19551 |
| FC_female_high | FC_male_medium | -1.44294 | 1.473495 | -0.98 | 0.3278 | -4.33559 | 1.44971 |
| FC_female_low | FC_female_medium | 2.37233 | 1.474396 | 1.61 | 0.1080 | -0.52209 | 5.26674 |
| FC_female_low | FC_male_high | 3.69979 | 1.910158 | 1.94 | 0.0531 | -0.05008 | 7.44965 |
| FC_female_low | FC_male_low | -1.19113 | 1.910158 | -0.62 | 0.5331 | -4.94100 | 2.55874 |
| FC_female_low | FC_male_medium | 2.31131 | 1.473495 | 1.57 | 0.1172 | -0.58133 | 5.20396 |
| FC_female_medium | FC_male_high | 1.32746 | 1.474396 | 0.90 | 0.3682 | -1.56696 | 4.22187 |
| FC_female_medium | FC_male_low | -3.56346 | 1.474396 | -2.42 | 0.0159* | -6.45787 | -0.66904 |
| FC_female_medium | FC_male_medium | -0.06101 | 0.834462 | -0.07 | 0.9417 | -1.69916 | 1.57713 |
| FC_male_high | FC_male_low | -4.89091 | 1.910158 | -2.56 | 0.0106* | -8.64078 | -1.14105 |
| FC_male_high | FC_male_medium | -1.38847 | 1.473495 | -0.94 | 0.3463 | -4.28112 | 1.50417 |
| FC_male_low | FC_male_medium | 3.50244 | 1.473495 | 2.38 | 0.0177* | 0.60980 | 6.39509 |

***Streptococcus alactolyticus***

| **Feed conversion Gender** | **Estimate** | **Std Error** | **DF** | **Lower 95%** | **Upper 95%** | **Arithmetic Mean Estimate** | **N** |
| --- | --- | --- | --- | --- | --- | --- | --- |
| FC_female_high | 8.581803 | 2.0763884 | 753 | 4.5056050 | 12.658002 | 8.581803 | 50 |
| FC_female_low | 5.367097 | 2.0763884 | 753 | 1.2908986 | 9.443295 | 5.367097 | 50 |
| FC_female_medium | 6.547652 | 0.9088105 | 753 | 4.7635489 | 8.331756 | 6.547652 | 261 |
| FC_male_high | 13.989286 | 2.0763884 | 753 | 9.9130880 | 18.065485 | 13.989286 | 50 |
| FC_male_low | 7.280618 | 2.0763884 | 753 | 3.2044199 | 11.356817 | 7.280618 | 50 |
| FC_male_medium | 8.147349 | 0.9053484 | 753 | 6.3700419 | 9.924656 | 8.147349 | 263 |

| **Feed conversion Gender** | **-Feed conversion Gender** | **Difference** | **Std Error** | **t Ratio** | **Prob>\|t\|** | **Lower 95%** | **Upper 95%** |
| --- | --- | --- | --- | --- | --- | --- | --- |
| FC_female_high | FC_female_low | 3.21471 | 2.936457 | 1.09 | 0.2740 | -2.5499 | 8.9793 |
| FC_female_high | FC_female_medium | 2.03415 | 2.266567 | 0.90 | 0.3698 | -2.4154 | 6.4837 |
| FC_female_high | FC_male_high | -5.40748 | 2.936457 | -1.84 | 0.0659 | -11.1721 | 0.3571 |
| FC_female_high | FC_male_low | 1.30119 | 2.936457 | 0.44 | 0.6578 | -4.4634 | 7.0658 |
| FC_female_high | FC_male_medium | 0.43445 | 2.265181 | 0.19 | 0.8480 | -4.0124 | 4.8813 |
| FC_female_low | FC_female_medium | -1.18056 | 2.266567 | -0.52 | 0.6026 | -5.6301 | 3.2690 |
| FC_female_low | FC_male_high | -8.62219 | 2.936457 | -2.94 | 0.0034* | -14.3868 | -2.8576 |
| FC_female_low | FC_male_low | -1.91352 | 2.936457 | -0.65 | 0.5148 | -7.6781 | 3.8511 |
| FC_female_low | FC_male_medium | -2.78025 | 2.265181 | -1.23 | 0.2201 | -7.2271 | 1.6666 |
| FC_female_medium | FC_male_high | -7.44163 | 2.266567 | -3.28 | 0.0011* | -11.8912 | -2.9921 |
| FC_female_medium | FC_male_low | -0.73297 | 2.266567 | -0.32 | 0.7465 | -5.1825 | 3.7166 |
| FC_female_medium | FC_male_medium | -1.59970 | 1.282806 | -1.25 | 0.2128 | -4.1180 | 0.9186 |
| FC_male_high | FC_male_low | 6.70867 | 2.936457 | 2.28 | 0.0226* | 0.9441 | 12.4733 |
| FC_male_high | FC_male_medium | 5.84194 | 2.265181 | 2.58 | 0.0101* | 1.3951 | 10.2888 |
| FC_male_low | FC_male_medium | -0.86673 | 2.265181 | -0.38 | 0.7021 | -5.3136 | 3.5801 |

***Enterococcus faecium***

| **Feed conversion Gender** | **Estimate** | **Std Error** | **DF** | **Lower 95%** | **Upper 95%** | **Arithmetic Mean Estimate** | **N** |
| --- | --- | --- | --- | --- | --- | --- | --- |
| FC_female_high | 2.9013568 | 0.76313182 | 753 | 1.403238 | 4.3994757 | 2.9013568 | 50 |
| FC_female_low | 0.2713622 | 0.76313182 | 753 | -1.226757 | 1.7694810 | 0.2713622 | 50 |
| FC_female_medium | 1.1775942 | 0.33401372 | 753 | 0.521885 | 1.8333030 | 1.1775942 | 261 |
| FC_male_high | 2.3984280 | 0.76313182 | 753 | 0.900309 | 3.8965469 | 2.3984280 | 50 |
| FC_male_low | 1.3550441 | 0.76313182 | 753 | -0.143075 | 2.8531629 | 1.3550441 | 50 |
| FC_male_medium | 1.7231673 | 0.33274128 | 753 | 1.069956 | 2.3763781 | 1.7231673 | 263 |

| **Feed conversion Gender** | **-Feed conversion Gender** | **Difference** | **Std Error** | **t Ratio** | **Prob>\|t\|** | **Lower 95%** | **Upper 95%** |
| --- | --- | --- | --- | --- | --- | --- | --- |
| FC_female_high | FC_female_low | 2.62999 | 1.079231 | 2.44 | 0.0150* | 0.51133 | 4.74865 |
| FC_female_high | FC_female_medium | 1.72376 | 0.833028 | 2.07 | 0.0389* | 0.08843 | 3.35910 |
| FC_female_high | FC_male_high | 0.50293 | 1.079231 | 0.47 | 0.6413 | -1.61573 | 2.62159 |
| FC_female_high | FC_male_low | 1.54631 | 1.079231 | 1.43 | 0.1523 | -0.57235 | 3.66497 |
| FC_female_high | FC_male_medium | 1.17819 | 0.832518 | 1.42 | 0.1574 | -0.45614 | 2.81252 |
| FC_female_low | FC_female_medium | -0.90623 | 0.833028 | -1.09 | 0.2770 | -2.54157 | 0.72910 |
| FC_female_low | FC_male_high | -2.12707 | 1.079231 | -1.97 | 0.0491* | -4.24573 | -0.00841 |
| FC_female_low | FC_male_low | -1.08368 | 1.079231 | -1.00 | 0.3156 | -3.20234 | 1.03498 |
| FC_female_low | FC_male_medium | -1.45181 | 0.832518 | -1.74 | 0.0816 | -3.08614 | 0.18253 |
| FC_female_medium | FC_male_high | -1.22083 | 0.833028 | -1.47 | 0.1432 | -2.85617 | 0.41450 |
| FC_female_medium | FC_male_low | -0.17745 | 0.833028 | -0.21 | 0.8314 | -1.81278 | 1.45788 |
| FC_female_medium | FC_male_medium | -0.54557 | 0.471468 | -1.16 | 0.2476 | -1.47112 | 0.37997 |
| FC_male_high | FC_male_low | 1.04338 | 1.079231 | 0.97 | 0.3340 | -1.07528 | 3.16204 |
| FC_male_high | FC_male_medium | 0.67526 | 0.832518 | 0.81 | 0.4176 | -0.95907 | 2.30959 |
| FC_male_low | FC_male_medium | -0.36812 | 0.832518 | -0.44 | 0.6585 | -2.00246 | 1.26621 |
